# Altering Residue 134 Confers an Increased Substrate Range of Alkylated Nucleosides to the *E. coli* OGT Protein

**DOI:** 10.3390/molecules22111948

**Published:** 2017-11-11

**Authors:** Nadia M. Schoonhoven, Derek K. O’Flaherty, Francis P. McManus, Lauralicia Sacre, Anne M. Noronha, M. Judith Kornblatt, Christopher J. Wilds

**Affiliations:** 1 Department of Chemistry and Biochemistry, Concordia University, Montréal, QC H4B 1R6, Canada; nadiaschoonhoven@hotmail.com (N.M.S.); derek_oflaherty@hotmail.com (D.K.O.); shent13@hotmail.com (F.P.M.); dlsacre@hotmail.com (L.S.); anne.noronha@concordia.ca (A.M.N.); 2 Howard Hughes Medical Institute, Department of Molecular Biology and Center for Computational and Integrative Biology, Massachusetts General Hospital, Boston, MA 02114, USA; 3 Institute for Research in Immunology and Cancer, Université de Montréal, Montréal, QC H3T 1J4, Canada

**Keywords:** Bioorganic molecules, modified oligonucleotides, DNA repair, substrate specificity, mutagenesis, homology modeling, DNA interstrand cross-link

## Abstract

*O*^6^-Alkylguanine-DNA alkyltransferases (AGTs) are proteins responsible for the removal of mutagenic alkyl adducts at the *O*^6^-atom of guanine and *O*^4^-atom of thymine. In the current study we set out to understand the role of the Ser134 residue in the *Escherichia coli* AGT variant OGT on substrate discrimination. The S134P mutation in OGT increased the ability of the protein to repair both *O*^6^-adducts of guanine and *O*^4^-adducts of thymine. However, the S134P variant was unable, like wild-type OGT, to repair an interstrand cross-link (ICL) bridging two *O*^6^-atoms of guanine in a DNA duplex. When compared to the human AGT protein (hAGT), the S134P OGT variant displayed reduced activity towards *O*^6^-alkylation but a much broader substrate range for *O*^4^-alkylation damage reversal. The role of residue 134 in OGT is similar to its function in the human homolog, where Pro140 is crucial in conferring on hAGT the capability to repair large adducts at the *O*^6^-position of guanine. Finally, a method to generate a covalent conjugate between hAGT and a model nucleoside using a single-stranded oligonucleotide substrate is demonstrated.

## 1. Introduction

The genetic information stored in DNA is under constant modification by endogenous and exogenous agents, including alkylating agents. Two highly mutagenic arrays of lesions induced by alkylating agents include *O*^6^-alkyl guanine and *O*^4^-alkyl thymine [1]. These modified bases, alkylated at the exocyclic oxygens, have altered hydrogen bonding patterns that can lead to errors in DNA replication by the action of DNA polymerases leading to the formation of point mutations [2]. These ensuing mutations in the genome can be extremely harmful to the cell and can be propagated during cell division, amplifying their occurrence.

Accordingly, cells have evolved repair systems to alleviate DNA damage. *O*^6^-alkylguanine-DNA alkyltransferases (AGTs), which are found in all kingdoms of life except plants, play an important role in maintaining the genomic code due to their ability to repair both *O*^6^-alkyl guanine and *O*^4^-alkyl thymine damage [3]. These alkyl adducts are transferred in a one-step process from either the *O*^6^-atom of guanine or the *O*^4^-atom of thymine to the active site cysteine of AGT, restoring the original nucleobase [3,4].

One class of chemotherapeutic agents commonly used in the treatment of cancers are bi-functional alkylating agents. These compounds can form mono-adducts, such as *O*^6^-alkylated guanine, but can also form interstrand cross-links (ICLs) due to the presence of a second reactive moiety on the compound [5]. ICL formation by these agents in aberrant cells is believed to be therapeutically important due to their extremely high cytotoxic nature as a result of inhibition of DNA strand separation during DNA replication and their relatively low mutagenic potential [6]. Unfortunately, the formation of the ICL adduct is not exclusive and mono-adduct formation does occur to a much greater extent [7]. The formation of mono-adducts occur upon hydrolysis of the second reactive moiety of the bi-functional agent, instead of another nucleophilic site on the DNA, creating a lesion with a terminal alcohol.

**Figure 1:**
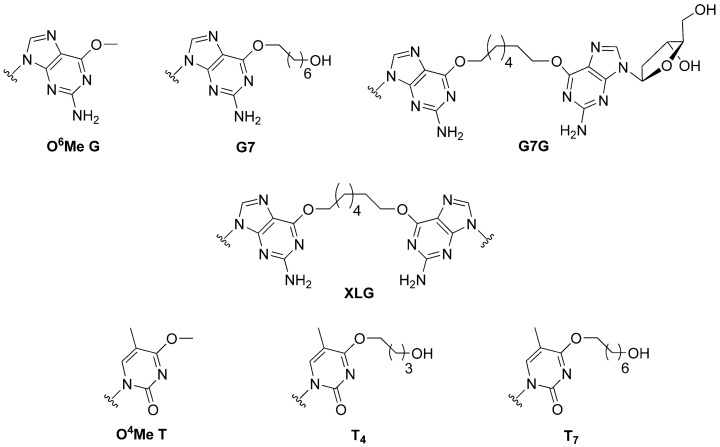
Structure of alkylated DNA modifications introduced into oligonucleotide substrates.

The mechanisms by which bi-functional alkylating agents and their corresponding lesions are processed are still under investigation, but do appear to require crosstalk between multiple repair machineries [8]. Understanding the role of DNA-alkyltransferases in this crosstalk consortium can enhance our knowledge of the processing of certain ICL and cross-linking compounds. The substrate library, shown in Figure 1, was created to verify three important roles these proteins could play in limiting the cytotoxicity and mutagenicity of bi-functional alkylating agents. That is, (*i*) Can DNA-alkyltransferases reduce off-target effects of bi-functional alkylating agents by removing mutagenic mono-adducts formed at the *O*^6^-atom of guanine or the *O*^4^-atom of thymine (**G7**, **T4** and **T7**)? (*ii*) Work by the Miller group has demonstrated that the processing of synthetically generated ICL DNA in human cell extracts generates DNA strands covalently linked to a single nucleoside by the ICL linker [9]; could such products be substrates for AGT mediated repair (**G7G**)? (*iii*) In the event where an ICL is fully formed, can both strands of DNA be de-alkylated by DNA alkyltransferases, restoring the DNA without the aid of other repair systems (**XLG**)?

Crystal structures of the human AGT (hAGT) in complex with DNA demonstrate that when bound by the protein, the alkyl appendage at both the *O*^6^-atom of purines or *O*^4^-atom of pyrimidines of modified DNA lies in a pocket formed by loop 135-144 [10,11]. This loop contains 3 proline residues at positions 138, 140, 144. Of these three prolines, Pro138 and Pro140 are important determinants of substrate specificity as illustrated by the decreased activity of the P140A and P138A hAGT variants for large alkyl substituents, such as the *O*^6^-benzyl group [12,13]. 

*E. coli* possesses two AGTs, Ada-C and OGT. Both have a preference for small substrates such as *O*^6^-methyl and ethyl guanine adducts and can efficiently repair these adducts at the *O*^4^-atom of thymine, unlike the human homolog [14]. In contrast to hAGT, *O*^6^-benzyl guanine is a poor substrate for OGT and is not acted upon by Ada-C. Coincidentally, Ada-C lacks both Pro138 and Pro140 residues. Mutating both Lys314 and Ala316 (corresponding to 138 and 140 in hAGT) to prolines imparts Ada-C with increased activity for large substrates [15]. OGT lies between Ada-C and hAGT as it possesses two of these three prolines but has serine, not proline, at position 134 (residue 140 in hAGT). Previous work by our group demonstrated that wild-type OGT was unable to repair the ICL DNA **XLG** while hAGT was able to repair this same substrate, highlighting major substrate differences between AGT homologs [16]. However, neither the P140K nor the P140A variants of hAGT repaired the ICL, proving the importance of Pro140 in hAGT mediated repair of ICL [16,17]. In addition, hAGT, OGT and Ada-C were unable to repair ICL between directly opposed thymidine residues at the *O*^4^-atoms *via* a butylene or heptylene linker [18]. 

We have previously shown that hAGT was capable of repairing intrastrand cross-linked (IaCL) DNA, both found in duplexes or as single-strand sequences [19,20]. Moreover, we showed that the presence or lack of the intra dimer phosphodiester group played an important role in the efficiency of hAGT mediated repair, with overall greater proficiency towards those IaCL lacking the phosphodiester linkage. This was attributed to the increased flexibility inherent to IaCL lacking the backbone phosphodiester group. To our surprise, no other tested AGTs, including OGT, were capable of efficient repair (<20%). Previous work from our group also showed an efficient method of covalently conjugating hAGT to DNA oligomers using either ICL [21] or IaCL DNA [22]. This methodology could not be used for OGT due to inefficient repair. As such, another objective set by this study was to elucidate a substrate scope for conjugating OGT to DNA substrates. Covalent conjugates of OGT and DNA could aid in the pursuit of understanding OGT’s substrate specificity. That is, no crystal structures of OGT have been published to date and perhaps these covalent complexes would crystallize more readily. It should be noted that an important part of the hAGT-mediated repair mechanism was derived from such covalent complexes [11]. 

The S134P OGT variant was generated by site-directed mutagenesis to assess the role of residue 134 on substrate discrimination. From the properties observed for the Ada-C double mutant and the P140 variants of hAGT we reasoned the S134P mutation in OGT would increase the substrate range of the protein. The impact of the S134P alteration on OGT function was analyzed through time course repair assays of the substrates in Figure 1 and comparing the ensuing rates with those of the wild-type OGT and hAGT.

## 2. Results

### 2.1. Syntheses and Oligonucleotide Substrate Preparation and Characterization

**Scheme 1:**
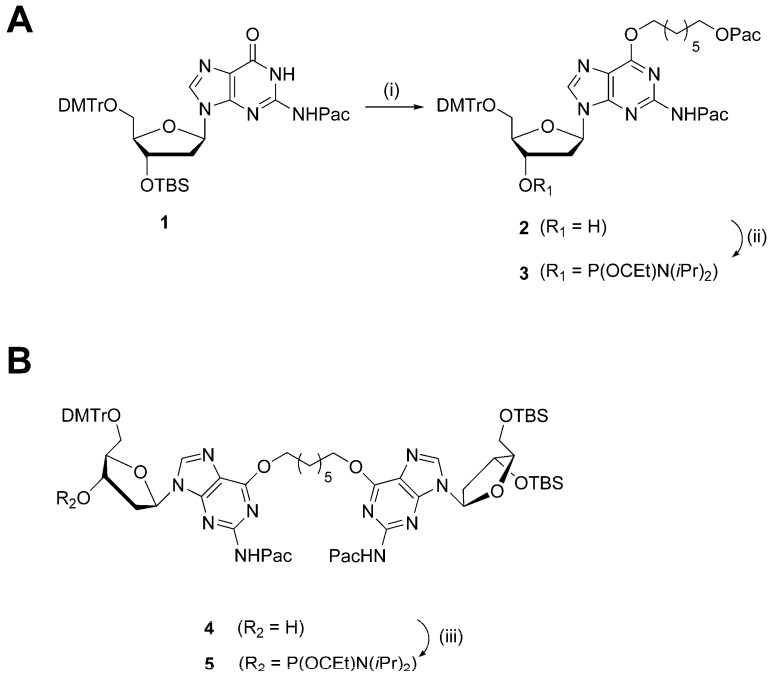
(**A**) Reagents and conditions: (i) 1. 7-hydroxyheptyl 2-phenoxyacetate (1.2 eqv.), PPh_3_ (1.2 eqv.), diisopropyl azodicarboxylate (1.2 eqv.), dioxane, 21 °C, 16 h; 2. TBAF (1 M in THF), THF, 21 °C, 30 min; (ii) *N*,*N*-diisopropylamino cyanoethyl phosphonamidic chloride (1.2 eqv.), N,N-diisopropylethylamine (1.5 eqv.), THF, 21 °C, 30 min. (**B**) Reagents and conditions: (iii) *N*,*N*-diisopropylamino cyanoethyl phosphonamidic chloride (1.2 eqv.), *N*,*N*-diisopropylethylamine (1.5 eqv.), THF, 21 °C, 30 min.

The syntheses of phosphoramidites **3** and **5**, in order to prepare the **G7** and **G7G** DNA probes, are shown in Scheme 1. Mono-adduct **2** was prepared in two steps from protected dG nucleoside **1** *via* a Mitsunobu coupling with 7-hydroxyheptyl 2-phenoxyacetate, followed by desilylation at the 3′-*O* functionality. The intermediate was phosphitylated using *N*,*N*-diisopropylamino cyanoethyl phosphonamidic chloride and Hünig’s base. The synthesis of dimer **4** has been previously described [20] and the free 3′ hydroxyl functionality was phosphitylated to produce the desired phosphoramidite **5**. The isolated phosphoramidites **3** and **5** were characterized by high-resolution mass spectrometry with the determined masses in agreement with the expected masses. Moreover, ^31^P NMR analysis was performed and revealed the presence of two sharp signals for each of the phosphoramidites in the region of 148.0–148.5 ppm, customary for these derivatives (see Supplementary Materials for NMR spectra).

Oligomers ***O*^6^Me G** and ***O*^4^Me T** were prepared by TriLink Biotechnologies (San Diego, CA, USA), whereas sequences **XLG**, **T4**, and **T7** were prepared according to previously published procedures [17,18]. Oligomers containing site-specific modifications were assembled by automated solid-phase synthesis (see the Supplementary Materials for sequence contexts). Two similar strategies were utilized to prepare **G7** and **G7G** DNA sequences. Both strategies required the use of phenoxyacetyl anhydride as the capping reagent during assembly, as to prevent undesired protecting group exchange at the *N*^2^-position of *O*^6^-alkylated-2′-deoxyguanosinyl inserts [23]. In both cases, phosphoramidites **3** and **5** were coupled with longer wait times (600 s as opposed to the standard 120 s) at a concentration of 0.15 M (0.1 M for standard phosphoramidites). Standard deprotection protocol was used to cleave and deprotect **G7** and **G7G** from the solid-support. The remnant 5′-*O* and 3′-*O*-TBS protecting groups of **G7G** were removed by additional treatment of the crude oligomer pellet with triethylamine trihydrofluoride. The **G7** and **G7G** sequences were purified using SAX-HPLC and corresponding purified DNA sequences were characterized *via* mass spectrometric analysis. The deconvoluted masses were in agreement with expected masses (see Supplementary Materials Figures S9 and S10).

**Figure 2:**
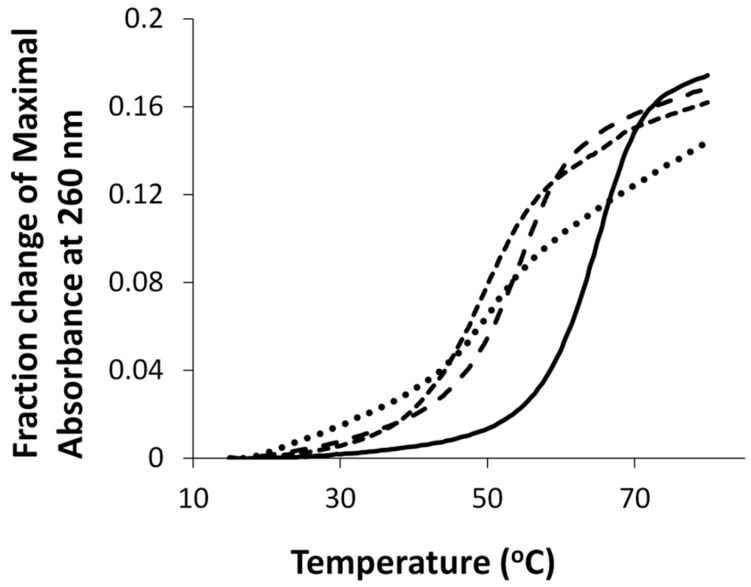
Hyperchromicity change (*A_260_*) versus temperature (°C) profiles of duplexes containing **G7** (•••), **G7G** (- - -), **O^6^Me G** (— — —), and unmodified **G** control DNA (—). *T_m_* values of duplexes containing **G7**, **G7G**, **O^6^Me G**, and unmodified **G** control DNA were 53 °C, 49 °C, 54 °C and 65 °C, respectively.

UV thermal denaturation of duplexes containing either **O^6^Me G**, **G7**, **G7G** or the unmodified control are shown in Figure 2. All duplexes exhibited monophasic sigmoidal transitions, with *T_m_* values of 54 °C, 53 °C, 49 °C, and 65 °C for duplexes containing **O^6^MeG**, **G7**, **G7G**, and the unmodified control, respectively.

**Figure 3:**
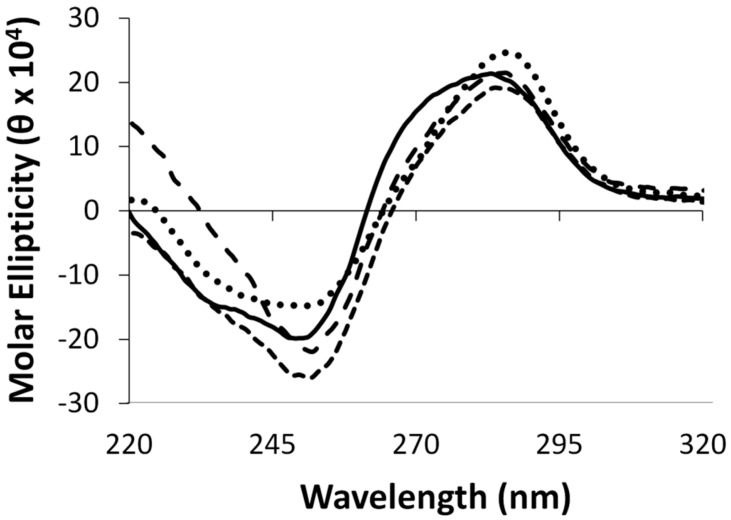
Circular dichroism spectra of duplexes containing G7 (•••), G7G (- - -), O^6^Me G (— — —), and unmodified G control DNA (—).

Circular dichroism spectroscopy was performed on duplexes containing the aforementioned modifications, in addition to the control duplex. All duplexes displayed characteristic signatures pertaining to the B-form DNA family with a positive signal centered around 280 nm, a crossover around 265 nm and a negative signal around 250 nm, as shown in Figure 3. The adducted duplexes revealed similar profiles to the unmodified duplexes suggesting minimal global structural distortions.

### 2.2. OGT Modeling

There are no 3-D structures of OGT. Therefore, before performing mutagenesis, we used homology modeling to explore the effects of a serine to proline mutation at position 134 of OGT. A homology model of OGT was constructed using the hAGT-*O*^6^-methyl-2′-deoxyguanosine co-crystal (PDB ID: 1t38) as the template [11]; **G7G** was then docked into the active site. **G7G**, whose coordinates were taken from a model generated by Fang, et al. [16], was designed to mimic the product resulting from the repair of the synthetic ICL DNA **XLG** [9]. The homology modeling and docking was repeated with the S134P OGT mutant for comparison.

**Figure 4:**
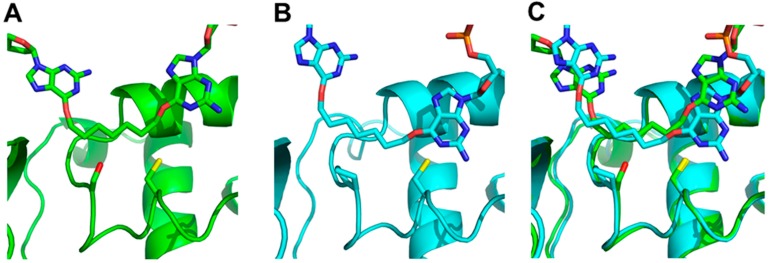
Docking of **G7G** to (**A**) OGT (green/dark) with Ser134 and Cys139 of OGT and the respective Pro140 and Cys145 of hAGT represented as sticks, (**B**) hAGT (cyan/light), (**C**) overlay of both hAGT and OGT. The *O*^6^-alkylated guanine undergoing repair is shown on the right of each panel.

The models (Figure 4) show that the Ser134 side chain (panel A) points into the loop that accommodates the alkyl adducts, thereby reducing the size of the active site relative to that of the human enzyme, as observed in the crystal structure. The observed distances between the active site thiolate anion, responsible for the proteins activity, and the α-carbon of the adduct were calculated to be: 2.99 Å for hAGT, 3.12 Å for the S134P OGT variant and 3.45 Å for OGT.

### 2.3. Protein Characterization 

The purified S134P OGT variant was analyzed by ESI-MS to confirm the identity of the protein; an observed mass of 20573.5 Da was obtained, which is in agreement with the calculated mass of 20573.8 Da. The variant repaired both **O^6^Me G** and **O^4^Me T** rapidly, where the reaction was over prior to the first time point of 15 s at room temperature, similar to OGT.

### 2.4. Enzymatic assays

#### 2.4.1. O^6^-Atom of Guanine

**Figure 5:**
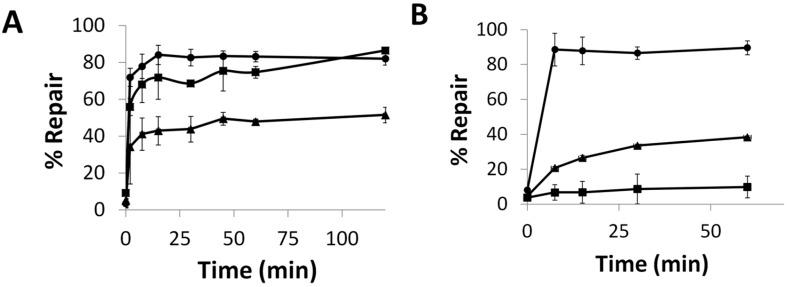
Time course repair of: (**A**) **G7** at 21 °C or (**B**) **G7G** at 37 °C, by OGT (■), the S134P variant of OGT (▲), and hAGT (●). See Figures S12 and S13 for denaturing PAGE of time course assays.

To evaluate hAGT repair of the alkylated nucleobases, the duplex was designed to contain a PvuII or BclI cleavage site—CAGCTG or TGATCA, respectively; incubation of the unmodified duplex with the respective restriction enzyme results in the 14-mer being cleaved to give shorter oligomers. When the central G (or T) in this sequence is modified, no cleavage occurs. If the O^6^-alkylguanine (or O^4^-alkylthymine) is repaired, cleavage can occur. This assay was inspired by an assay developed by Moser, et al. [24] hAGT, OGT and the S134P OGT variant were efficient at repairing **G7**. The repair of **G7** by these proteins was evaluated at room temperature and observed to occur rapidly (Figure 5A). hAGT repaired the **G7** adduct before the 15 s time point whereas OGT took approximately 15 min to reach the repair plateau, eventually reaching total repair. Introducing the S134P modification in OGT led to a protein whose repair efficiency for **G7** was lower compared to OGT, reaching a plateau of approximately 50% repair.

The repair of **G7G** was also examined (Figure 5B). The *O*^6^-heptylene linked 2′-deoxyguanosine nucleoside adduct reduced the ability of all three proteins to restore the DNA. Due to the reduced efficiency towards this modification observed by the two OGTs, the time course assay was carried out at 37 °C. OGT was virtually unable to repair the adduct with less than 10% repair observed in an hour. During the time period, its S134P variant achieved 40% repair. The variant was not as efficient as hAGT, which was able to achieve full repair within 7.5 min. 

**Figure 6:**
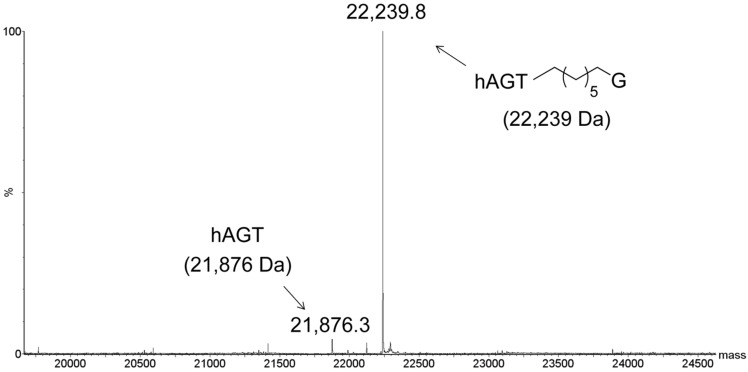
Identification of hAGT-DNA covalent complex by ESI-MS. Product identified from reaction of hAGT with single-stranded **G7G**. Values in brackets represent the calculated masses. No conjugation was observed for the reaction of OGT or OGT S134P with single-stranded **G7G** (data not shown).

To determine if the **G7G** probe could be utilized as a means of conjugating a monomer to AGTs, the repair was conducted on single-stranded **G7G** with the three AGT homologs. Due to the small mass shift for the alkyl transfer, analysis was conducted by LC-MS, where only hAGT displayed efficient covalent complex formation (Figure 6). Our approach has expanded the toolbox to generate hAGT conjugates using single-stranded oligomers containing a tethered nucleoside.

#### 2.4.2. O^4^-Atom of Thymine

**Figure 7:**
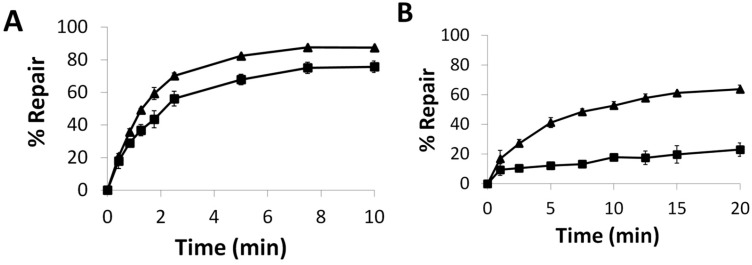
Time Course Repair of: (**A**) **T4** or (**B**) **T7** at 21 °C, by OGT (■) and the S134P variant of OGT (▲).

The influence of the S134P mutation in OGT was further analyzed by evaluating the repair of alkylation from the *O*^4^-atom of thymine. We have shown that hAGT is unable to repair **T4** and **T7** but was active towards ***O*^4^Me T** [18]. The S134P mutation in OGT conferred a slight increase in repair efficiency towards **T4** (Figure 7A). The effect of the mutation was amplified as the lesion increased in size, as observed for repair of **T7** (Figure 7B) where a 3-fold increase in repair was observed for the S134P variant of OGT over the native protein after 15 min.

### 2.5. Repair and Binding of ICL

Our repair assay results revealed the S134P variant of OGT, much like the wild-type, was unable to repair **XLG** (data not shown). The lack of repair of **XLG** by the S134P OGT variant indicates that the absence of Pro134 (Pro140 in hAGT) in OGT is not solely responsible for the inability of OGT to repair ICL.

**Figure 8:**
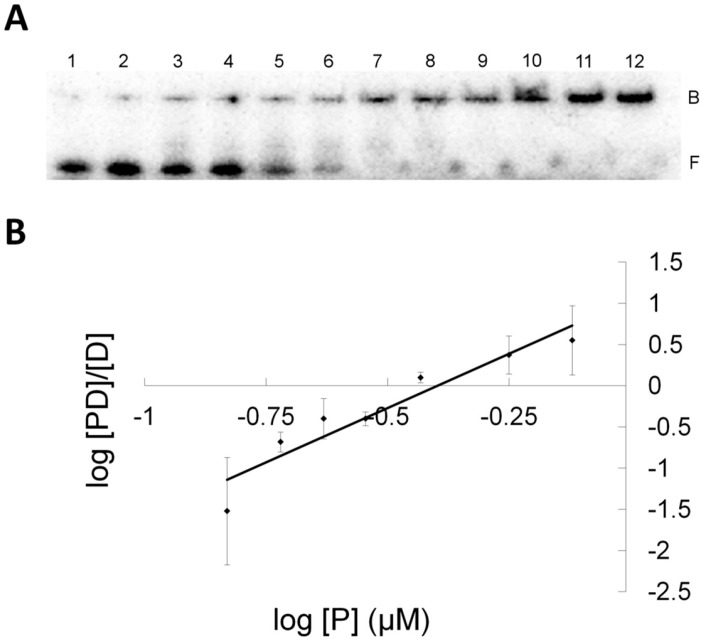
Electromobility shift assay of S134P OGT binding to **XLG** (**A**) EMSA gel of titration of **XLG** with S134P OGT. 0.5 nM **XLG** and increasing amounts of S134P OGT from 0.1 μM to 4 μM, from lane 1 to 12. Band B represent the bound and band F the free DNA species. (**B**) Hill plot representation for the linear range of log[PD]/[D] versus log[P] for S134P OGT and **XLG** (♦).

To address the cause for this lack of activity, EMSA were performed to obtain binding information for the DNA-protein complexes (Figure 8). The results shown in Table 1 indicates both OGT and its S134P variant were capable of binding **XLG** and did so with similar affinities implying the S134P alteration in OGT had no effect on substrate recognition. Interestingly, under our EMSA conditions, performed in the absence of NaCl, both OGT and its S134P variant bound the control DNA (having a G-C match whereas the G-(CH_2_)_7_-G cross-link is found in **XLG**) and **XLG** with higher affinity than hAGT suggesting that the lack of repair of the ICL was not caused by poor substrate interaction. Like hAGT, OGT and its S134P variant had slightly higher binding affinities for **XLG** than for the control sequence.

## 3. Discussion

DNA sequences **G7** and **G7G** were assembled, cleaved from the solid-support and deprotected with slight modifications to previously reported procedures. In the case of **G7G**, the remaining silyl protecting groups found at the 5′- and 3′-*O* of the dangling *O*^6^-alkylene-dG residue were removed by triethylamine trihydrofluoride treatment of the crude solid material. Sonication of the reaction mixture was initially carried out (two 15 s treatments) to assist the reaction, presumably by breaking up solid particles to allow full contact of the oligomer to the fluoride reagent. Purification of **G7** and **G7G** was achieved by SAX-HPLC, which generally exhibits better recoveries compared to purification by PAGE in our hands. DNA oligonucleotides were characterized using mass spectrometry, which revealed deconvoluted masses of **G7** and **G7G** in agreement with the expected masses (shown in the Supplementary Materials Figures S9 and S10). DNA oligomer syntheses were performed on scales amenable for biochemical characterization and assays, as well as structural studies such as X-ray crystallography and NMR spectroscopy.

The DNA duplexes containing the modifications displayed reduced stability, by approximately 12–16 °C, relative to the unmodified DNA duplexes. Interestingly, the presence of a longer hydroxyheptylene adduct (**G7**) did not significantly change the *T_m_* of the duplex relative to those containing an ***O*^6^Me G**. The presence of a tethered nucleoside (**G7G**), however, revealed a lowering in *T_m_* in comparison to the other G-adducted DNA duplexes. Similar reductions in *T_m_* have been observed for duplexes containing ***O*^4^Me T**, **T4** and/or **T7** (with an approximately 10 °C decrease for T-adducted DNA duplexes relative to the control) [18].

Circular dichroism profiles revealed no large structural perturbations for duplexes containing **O^6^Me G**, **G7** and **G7G** relative to the corresponding control. The profiles were in agreement with signatures of the B-family DNA. Moreover, basic geometry optimization was performed for duplexes containing **G7** and **G7G** (shown in Supplementary Materials Figure S11). It should be noted that DNA duplex structure containing an *O*^6^Me G insert has previously been reported using a combination of molecular dynamics and high-field NMR experiments [25,26]. Both lesions in **G7** and **G7G** were found to protrude into the major groove of the duplex, with increased distortions observed in the vicinity of the adducted G. These distortions were more pronounced for **G7G**. The molecular models suggest that the presence of the lesions do not greatly perturb the global structure of the duplex, which was in agreement with circular dichroism profiles observed for **G7** and **G7G**. Interestingly, the **G7G** model depicted the tethered nucleoside as being solvent exposed. High resolution structures of duplexes containing these modifications are currently under investigation. 

Steric effects play a substantial role in AGT-mediated repair of alkylation at both the *O*^6^-atom of guanine and *O*^4^-atom of thymine. Generally, as the adduct increases in size the rate of repair decreases. Much of the knowledge of AGT proteins have stemmed from work with the human homolog and research involving the *E. Coli* OGT are sparse.

To understand OGTs limited substrate range compared to the human homolog molecular modeling as well as docking studies were utilized (Figure 4). Our in silico analysis suggested the presence of Ser134 instead of proline not only decreased the size of the active site but also rendered a portion of the active site polar. This region is non-polar in hAGT and stacks against the adducts at both the *O*^6^-atom of guanine and *O*^4^-atom of pyrimidines by a hydrophobic interaction [10,11]. This favorable hydrophobic interaction between adduct and protein would therefore be missing in OGT. These different properties between serine and proline resulted in the α-carbon of the linker from **G7G** being further away from the active site thiolate anion in OGT. The replacement of this serine by proline partially restored the position of the substrate relative to the thiolate anion (data not shown). The docking results suggested the S134P OGT variant would have increased repair activity for **G7G** and more generally, improved repair activity for larger adducts at the *O*^6^-atom of guanine and even at the *O*^4^-atom of thymine compared to wild-type.

Preparation and characterization of the S134P variant of OGT was achieved and *in vitro* repair assays performed to verify the validity of our in silico studies. Our time course assays revealed the S134P variant of OGT had improved repair capabilities for larger substrate (**G7G**) at both the *O*^6^-atom of guanine and *O*^4^-atom of thymine compared to the wild-type, except for the **G7** adduct. The effect of the amino acid alteration was amplified as the lesions increased in size, as exemplified by the ability of the S134P variant to repair **G7G** where its wild-type equivalent was refractory towards such damage. A similar dealkylation propensity was observed for the *O*^4^-atom of thymine where the rate of repair of the butan-4-ol lesion was virtually identical between OGT proteins while the longer heptan-7-ol was repaired 3 times faster by the variant. The observed increase in the efficiency of repair of the larger adducts for the S134P variant of OGT, irrelevant of the alkylated atom, suggests both the adducts at the *O*^6^-atom of guanine and *O*^4^-atom of thymine are similarly placed in the active site of OGT and interact with residue 134 in some fashion. The presence of proline at residue 134 of OGT appears to play the same role as it does in hAGT where Pro140 increases the size of the active site of the protein allowing it to accommodate larger lesions for repair.

hAGT was more proficient than both OGTs at removing the lesions at the *O*^6^-atom of guanine, but was unable to repair the butan-4-ol and heptan-7-ol adducts at the *O*^4^-atom of thymine. Such results were anticipated since hAGT is known to repair **XLG** and is virtually inactive against *O*^4^-methyl thymidine. Moreover, *in vivo*, overexpression of this protein increases the toxicity of *O*^4^-methyl thymidine by shielding it from the NER machinery, which can repair this damage [1,16,27,28]. An interesting finding from this study stems from the efficient conjugation of hAGT to a single nucleoside, bridged by an alkylene tether. This novel conjugation approach may find applications in biotechnology as functional probes in a similar fashion as previously described [29,30,31]. 

Despite promising results with the S134P variant of OGT with the mono-adducts the variant fell short with **XLG**, which was not repaired. The EMSA results indicated the OGT proteins bound **XLG** with higher affinity than hAGT, which is capable of repairing such a lesion. Clearly, OGT and its S134P variant were able to interact with **XLG** with reasonable affinities, which indicated the proteins recognized the ICL DNA. Both OGT proteins and hAGT were able to discriminate between native DNA and ICL DNA as observed by the decreased dissociation constant (*K_d_*) for the ICL DNA. Hence, substrate recognition by OGT and its S134P variant was not the cause for these proteins inability to repair the ICL. The model of hAGT bound to the ICL substrate, constructed by Fang, et al. [16] involves extensive disruption of the double stranded structure. Moreover, the X-ray structures of hAGT bound to DNA show binding produces several changes in the DNA, including flipping out of the modified nucleotide from the DNA duplex, widening of the minor groove and bending of the DNA [10,32]. It is possible that neither OGT nor the S134P variant are able to produce the necessary changes in the DNA. Thus these enzymes show tighter binding than hAGT to the ICL substrate because they are not using binding energy to produce conformation changes in the substrate. Binding is tight, but non-productive.

The EMSA results revealed variations in Hill factor between hAGT and OGT binding to **XLG**. The Hill factor, which can be correlated with the stoichiometry of binding, indicated OGT and its S134P variant bound **XLG** with a stoichiometry between 3–4 proteins while hAGT bound the DNA duplex with a stoichiometry of 2. This difference in stoichiometry of binding is a clear indicator that the two homologs do not interact with **XLG** in a similar fashion. 

OGT shows a vast advantage at repairing *O*^4^-alkyl thymine lesions when compared to the human homolog. Attempts to generate hAGT variants capable of repairing *O*^4^-methyl thymidine more rapidly have been undertaken with appreciable gains [33,34,35]. However, even the most active variant is still 92 times slower than OGT. OGT, and more notably the S134P variant, are superior to any engineered protein in regards to repair of *O*^4^-thymidinyl alkylation damage. The increased substrate range provided by the S134P alteration further exemplifies the multifaceted role of OGT. This protein is not limited to small adducts, as once believed. 

Understanding the mechanistic and structural basis for OGTs ability to repair *O*^6^-alkyl guanine and *O*^4^-alkyl thymine with similar ease could hold valuable information regarding hAGT function, which may have a use in gene therapy and biotechnology. Imparting hAGT with OGT properties has the potential to limit some side effects developed during chemotherapeutic regimens due to OGTs ability to remove lesions, such as **G7**, **T4** and **T7**, while being inert to ICL, which are attributed to the success of bifunctional alkylating agents. Furthermore, due to the recent optimistic outcome of phase 1 clinical trials of carmustine used in combination with *O*^6^-benzylguanine, the development of *O*^6^-benzylguanine resistant hAGT variants for use in therapy to protect non-targeted cells has accrued much interest [36]. These resistant DNA-alkyltransferases, of which OGT is a part of, can be employed in selected tissues to impart them with alkyltransferase activity even in the presence of the inhibitor. Conferring the repair properties of OGT to the human homolog is a good avenue to pursue for engineering hAGT proteins that could be used during combinational therapy.

## 4. Materials and Methods

Please refer to Supplementary Materials for experimental details concerning the small molecule synthesis and characterization, modified oligonucleotide assembly, UV thermal denaturation experiments, circular dichroism spectroscopy, nucleic acid molecular modeling, OGT modeling, protein over-expression and purification, mutagenesis, enzymatic assays, binding assays, and identification of the hAGT covalent conjugate using **G7G** ssDNA.

## Figures and Tables

**Table 1:** *K_d_* values of AGT-DNA interactions.

Protein	*K_d_* (µM) Control (G-C)	*K_d_* (µM) XLG
C145S	10.61 ± 0.74	1.37 ± 0.01
OGT	1.23 ± 0.25	0.41 ± 0.02
S134P OGT	0.86 ± 0.10	0.36 ± 0.02

## Supplementary Materials

Please refer to supplementary materials available online, for ^1^H, ^13^C, and ^31^P NMR spectra, ESI MS spectra of **G7** and **G7G**, geometry optimized molecular models of **G7** and **G7G**, time course repair PAGE.
