# Supplementary material for: Altering Residue 134 Confers an Increased Substrate Range of Alkylated Nucleosides to the E. coli OGT Protein
Source: Molecules. 2017 Nov 11;22(11):1948. doi: 10.3390/molecules22111948 (PMC6150290; doi:10.3390/molecules22111948)
Supplement: Supplementary file 1 [file molecules-22-01948-s001.pdf]

Supplementary Materials

Nadia M. Schoonhoven[1‡], Derek K. O'Flaherty[1,2‡], Francis P. McManus[1,3‡], Lauralicia Sacre[1], Anne M. Noronha[1], M. Judith Kornblatt[1,*], and Christopher J. Wilds[1,*]

[1]Department of Chemistry and Biochemistry, Concordia University, Montréal, Québec, H4B 1R6, Canada

[2]Howard Hughes Medical Institute, Department of Molecular Biology and Center for Computational and Integrative Biology, Massachusetts General Hospital, Boston, Massachusetts, 02114, United States

[3]Institute for Research in Immunology and Cancer, Université de Montréal, Montréal, Québec, H3T 1J4, Canada

‡ These authors contributed equally

Contents **Page**

Supplementary Methods

Synthesis and characterization of nucleosides and oligonucleotides **S-3**
UV thermal denaturation **S-7**
Circular dichroism (CD) spectroscopy **S-7**
Nucleic acid molecular modeling **S-8**
OGT modeling **S-8**
Protein expression and purification **S-8**
Mutagenesis **S-8**
Enzymatic Assays **S-9**
Binding Assays **S-9**
Identification of repair product covalent conjugate from hAGT-mediated repair of **G7G** ssDNA **S-9**

Supplementary Figures

Supplementary Figure S1 - 500 MHz ^{1}H NMR spectrum of compound (**2**) (in $CDCl_3$) **S-11**
Supplementary Figure S2 - 125.7 MHz ^{13}C NMR spectrum of compound (**2**) (in $CDCl_3$) **S-12**
Supplementary Figure S3 - 500 MHz ^{1}H NMR spectrum of compound (**3**) (in d_6-acetone) **S-13**
Supplementary Figure S4 - 125.7 MHz ^{13}C NMR spectrum of compound (**3**) (in d_6-acetone) **S-14**
Supplementary Figure S5 - 202.3 MHz ^{31}P NMR spectrum of compound (**3**) (in d_6-acetone) **S-15**
Supplementary Figure S6 - 500 MHz ^{1}H NMR spectrum of compound (**5**) (in d_6-acetone) **S-16**
Supplementary Figure S7 - 125.7 MHz ^{13}C NMR spectrum of compound (**5**) (in d_6-acetone) **S-17**
Supplementary Figure S8 - 202.3 MHz ^{31}P NMR spectrum of compound (**5**) (in d_6-acetone) **S-18**
Supplementary Figure S9 - ESI MS spectrum of oligonucleotide **G7** **S-19**
Supplementary Figure S10 - ESI MS spectrum of oligonucleotide **G7G** **S-20**
Supplementary Figure S11 - Molecular models of unmodified control duplex (**G**) and duplexes containing **G7**, and **G7G** that were geometry optimized using the AMBER forcefield **S-21**

**Supplementary Figure S12 -**	Time course repair gel of duplexes containing **G7** by (**A**) hAGT, (**B**) OGT (**C**) S134P OGT	**S-23**
**Supplementary Figure S13 -**	Time course repair gel of duplexes containing **G7G** by (**A**) hAGT, (**B**) OGT (**C**) S134P OGT	**S-23**
**Supplementary References**		**S-24**

Synthesis and characterization of nucleosides and oligonucleotides

General

5'-*O*-(4,4'-Dimethoxytrityl)-N^2-phenoxyacetyl-2'-deoxyguanosine, and N,N-diisopropylaminocyanoethyl phosphonamidic chloride were purchased from ChemGenes, Inc. (Wilmington, MA). Protected dG monomer **1**[1] and dimer **4**[2] were prepared according to previously reported procedures. 5'-*O*-Dimethoxytrityl-2'-deoxyribonucleoside-3'-*O*-(β-cyanoethyl-N,N-diisopropyl)phosphoramidites and protected 2'-deoxyribonucleoside-CPG supports were purchased from Glen Research (Sterling, Virginia). All other chemicals and solvents were purchased from the Aldrich Chemical Company (Milwaukee, WI) or EMD Chemicals Inc. (Gibbstown, NJ). Flash column chromatography was performed using silica gel 60 (230-400 mesh) purchased from Silicycle (Quebec City, QC). Thin layer chromatography (TLC) was carried out with precoated TLC plates (Merck, Kieselgel 60 F_{254}, 0.25 mm) purchased from EMD Chemicals Inc. (Gibbstown, NJ). NMR spectra were recorded on a Varian 500 MHz NMR spectrometer at room temperature. ^{1}H NMR spectra were recorded at a frequency of 500.0 MHz and chemical shifts were reported in parts per million (ppm) downfield from tetramethylsilane. ^{13}C NMR spectra (^{1}H decoupled) were recorded at a frequency of 125.7 MHz and chemical shifts were reported in ppm with tetramethylsilane as a reference. ^{31}P NMR spectra (^{1}H decoupled) were recorded at a frequency of 202.3 MHz and chemical shifts were reported in ppm with H_3PO_4 used as an external standard. High resolution mass spectrometry of modified nucleosides were obtained using an 7T-LTQ FT ICR instrument (Thermo Scientific), at the Concordia University Centre for Structural and Functional Genomics. The mass spectrometer was operated in full scan, positive ion detection mode. ESI mass spectra for oligonucleotides were obtained at the Concordia University Centre for Biological Applications of Mass Spectrometry using a Micromass Qtof2 mass spectrometer (Waters) equipped with a nanospray ion source. The mass spectrometer was operated in full scan, negative ion detection mode. Ampicillin, isopropyl β-D-thiogalactopyranoside (IPTG), and most other biochemical reagents as well as polyacrylamide gel materials were purchased from Bioshop Canada Inc (Burlington, ON). Ni-NTA Superflow Resin was purchased from Qiagen (Mississauga, ON). Complete, Mini, EDTA-free Protease Inhibitor Cocktail Tablets were obtained from Roche (Laval, QC). Nitro-cellulose filters (0.20 µm) were obtained from Millipore. XL-10 Gold and Bl21(DE3) *E. coli* cells were obtained from Stratagene (Cedar Creek, TX). T4 polynucleotide kinase (PNK) was obtained from Fermentas (Burlington, ON). [γ-^{32}P]ATP was purchased from PerkinElmer (Woodbridge, ON). Phusion Polymerase was obtained from New England Biolabs (Ipswich, MA). DNA primers for site directed mutagenesis and cloning were purchased from Biocorp (Montreal, QC).

Chemical synthesis of nucleosides

5'-O-(4,4'-dimethoxytrityl)-N^2-phenoxyacetyl-O^6-(7-phenoxyacetyloxyheptylene)-2'-deoxyguanosine (**2**)

To a solution of **1** (0.62 g, 0.78 mmol), 7-hydroxyheptyl 2-phenoxyacetate (0.25 g, 0.94 mmol), and Ph_3P (0.25 g, 0.94 mmol) in anhydrous dioxane (5.0 mL) was added DIAD (0.18 mL, 0.94 mmol) dropwise while stirring. After 16 h, the solvent was removed in vacuo and the content taken up in DCM (50 mL), then washed with a saturated aqueous solution of $NaHCO_3$ (2 x 50 mL) followed with brine (50 mL). The

solvent was dried over anhydrous Na_2SO_4 (~ 4 g), decanted and then evaporated to afford a yellow gum. To this intermediate in THF (5 mL) was added TBAF (1 M in THF) (1.2 mL, 1.2 mmol) under stirring. After 20 min, the solvent was removed in vacuo and the content taken up in DCM (50 mL), then washed with a saturated aqueous solution of $NaHCO_3$ (2 x 50 mL) followed with brine (50 mL). The solvent was dried over anhydrous Na_2SO_4 (~ 4 g), decanted and then evaporated to afford a yellow gum. The product was purified *via* flash column chromatography using MeOH : DCM (1 % → 5 %) as eluent to afford 0.58 g (81 % over two steps) of a colorless foam. R_f (SiO_2 TLC):0.46 MeOH : DCM (5 %). $\lambda_{max(MeCN)}$ = 269 nm. ^1H NMR (500 MHz, $CDCl_3$, ppm): 8.76 (bs, 1H, NH), 8.02 (s, 1H, H8), 7.40-7.15 (m, 13H, Ar), 7.07-7.04 (m, 1H, Ar), 7.00-6.96 (m, 3H, Ar), 6.91-6.89 (m, 2H, Ar), 6.78-6.76 (m, 4H, Ar), 6.63 (dd, 1H, H1', *J* = 6.5 Hz), 4.80 (m, 1H, H3'), 4.66-4.62 (m, 4H, 2 x $PhOCH_2$), 4.56 (t, 2H, $ArOCH_2$, *J* = 6.5 Hz), 4.23-4.18 (m, 3H, H4', CH_2OOC), 3.74 (s, 6H, 2 x OCH_3), 3.45 (dd, 1H, H5', *J* = 4.5, 10.5 Hz), 3.34 (dd, 1H, H5'', *J* = 4.5, 10.5 Hz), 2.74 (m, 1H, H2'), 2.63 (m, 1H, H2''), 1.87 (m, 2H, CH_2), 1.65 (m, 2H, CH_2), 1.49 (m, 2H, CH_2), 1.40-1.32 (m, 4H, 2 x CH_2). ^{13}C NMR (125.7 MHz, $CDCl_3$, ppm): 169.06, 161.18, 158.48, 157.80, 157.09, 152.35, 150.93, 144.57, 140.24, 135.77, 135.69, 130.03, 130.00, 129.82, 129.52, 128.12, 127.82, 126.85, 122.38, 121.68, 118.96, 114.93, 114.61, 113.12, 86.51, 86.48, 84.15, 72.60, 67.91, 67.73, 65.34, 65.32, 64.12, 55.16, 40.74, 29.68, 28.88, 28.71, 28.44, 25.81, 25.68. IR (thin film); ν_{max} (cm^{-1}) = 3409, 3060, 2932, 2856, 2362, 2336, 1757, 1729, 1607, 1509, 1496, 1443, 1381, 1250, 1175, 1085, 830, 755, 735. HRMS (ESI-MS) *m/z* calculated for $C_{54}H_{57}N_5NaO_{11}^+$ 974.3947: found 974.3948 $[M+Na]^+$

3'-O-(2-cyanoethoxy(diisopropylamino)-phosphino)-5'-O-(4,4'-dimethoxytrityl)-N^2-phenoxyacetyl-O^6-(7-phenoxyacetyloxyheptylene)-2'-deoxyguanosine (**3**)

To a solution of **2** (0.28 g, 0.18 mmol) and DIPEA (60 µL, 0.35 mmol) in THF (1.8 mL) was added Cl-P(OCEt)(N*i*Pr_2) (60 µL, 0.27 mmol) and the reaction was allowed to stir at room temperature. After 30 min, the solvent was removed in vacuo and the crude was taken up in EtOAc (50 mL), then washed with a saturated aqueous solution of $NaHCO_3$ (2 x 40 mL) followed with brine (35 mL). The solvent was dried over anhydrous Na_2SO_4 (~ 1 g), decanted and then evaporated to afford a yellow gum. The crude product was purified *via* flash column chromatography using EtOAc (with 0.1% v/v NEt_3) as the eluent to afford 0.12 g (57 %) of a colorless foam. R_f (SiO_2 TLC): 0.84, 0.89 EtOAc. $\lambda_{max(MeCN)}$ = 269 nm. ^1H NMR (500MHz, d_6-acetone, ppm): 9.27 (bs, 1H, NH), 8.22-8.21 (m, 1H, H8), 7.44-7.41 (m, 2H, Ar), 7.34-7.14 (m, 11H, Ar), 7.04-6.90 (m, 6H, Ar), 6.80-6.73 (m, 4H, Ar), 6.51-6.48 (m, 1H, H1'), 5.06-5.05 (m, 2H, $PhOCH_2$), 5.00-4.89 (m, 1H, H3'), 4.71 (s, 2H, $PhOCH_2$), 4.60-4.56 (m, 2H, $ArOCH_2$), 4.33-4.24 (m, 1H, H4'), 4.15 (t, 2H, CH_2COO, *J* = 6.5 Hz), 3.91-3.50 (m, 11H, H5', 2 x OCH_3, 2 x NCH, $POCH_2$), 3.40-3.34 (m, 1H, H5''), 3.21-3.14 (m, 1H, H2'), 2.75-2.55 (m, 3H, H2'', CH_2CN), 1.89-1.83 (m, 2H, CH_2), 1.66-1.61 (m, 2H, CH_2), 1.51-1.45 (m, 2H, CH_2), 1.43-1.33 (m, 4H, CH_2CH_2), 1.21-1.17 (m, 9H, 3 x CH_3), 1.13-1.12 (m, 3H, CH_3). ^{13}C NMR (125.7 MHz, d_6-acetone, ppm): 169.99, 168.63, 166.72, 161.00, 158.63, 158.57, 158.21, 158.20, 152.67, 152.62, 151.63, 151.59, 145.28, 145.27, 141.11, 141.04, 135.95, 135.88, 135.87, 135.83, 130.13, 130.08, 129.99, 129.96, 129.47, 129.40, 128.12, 128.06, 127.56, 126.56, 126.54, 121.27, 121.24, 121.21, 118.72, 118.67, 118.14, 117.98, 117.03, 114.74, 114.47, 112.88, 112.87, 112.84, 112.83, 86.15, 86.12, 86.06, 86.05, 85.92, 85.87, 84.69, 84.62, 74.28, 74.14, 73.93, 73.80, 67.92, 67.18, 64.80, 64.57, 64.19, 61.90, 61.86, 59.65, 58.73, 58.67, 58.58, 58.52, 54.61, 54.60, 54.59, 47.31, 47.28, 43.13, 43.03, 38.43, 38.39, 28.72, 28.59, 28.39, 25.69, 25.56, 24.05, 24.02, 23.99, 23.97, 21.93, 21.91, 20.44, 20.42,

19.97, 19.90, 19.84, 19.78, 18.96, 18.88, 13.64. ^{31}P NMR (202.3 MHz, d_6-acetone, ppm): 148.44, 148.22. IR (thin film); ν_{max} (cm^{-1}) = 3410, 2968, 2934, 2863, 2365, 2334, 1712, 1606, 1510, 1495, 1465, 1419, 1365, 1249, 1176, 1083, 1032, 977, 830, 755, 691. HRMS (ESI-MS) *m/z* calculated for $C_{63}H_{74}N_7NaO_{12}P^+$ 1174.5025: found 1174.5072 $[M+Na]^+$.

1- {O^6-[3'-O-(2-cyanoethoxy(diisopropylamino)-phosphino)-5'-O-(4,4'-dimethoxytrityl)-N^2-phenoxyacetyl-2'-deoxyguanidinyl]}-7-{O^6-[3',5'-O-bis(tert-butyldimethylsilyl)-N^2-phenoxyacetyl-2'-deoxyguanidinyl]}-heptane (**5**)

To a solution of **4** (0.20 g, 0.14 mmol) and DIPEA (46 µL, 0.26 mmol) in THF (1.5 mL) was added Cl-P(OCEt)(N*i*Pr_2) (46 µL, 0.21 mmol) and the reaction was allowed to stir at room temperature. After 30 min, the solvent was removed in vacuo and the crude was taken up in EtOAc (50 mL), then washed with a saturated aqueous solution of $NaHCO_3$ (2 x 40 mL) followed with brine (35 mL). The solvent was dried over anhydrous Na_2SO_4 (~ 1 g), decanted and then evaporated to afford a yellow gum. The crude product was purified *via* flash column chromatography using EtOAc (with 0.1% v/v NEt_3) as the eluent to afford 0.18 g (79 %) of a colorless foam. R_f (SiO_2 TLC): 0.92, 0.95 EtOAc. $\lambda_{max(MeCN)}$ = 269 nm. ^{1}H NMR (500MHz, d_6-acetone, ppm): 9.39 (bs, 1H, NH), 9.27 (bs, 1H, NH), 8.24 (s, 1H, H8b), 8.20 (m, 1H, H8a), 7.43-7.38 (m, 2H, Ar), 7.32-7.26 (m, 8H, Ar), 7.22-7.14 (m, 3H, Ar), 7.02-6.94 (m, 6H, Ar), 6.80-6.71 (m, 4H, Ar), 6.51-6.47 (m, 1H, H1'a), 6.43 (t, 1H, H1'b, J = 6.5 Hz), 5.05-5.04 (m, 4H, 2 x $PhOCH_2$), 5.00-4.88 (m, 1H, H3'a), 4.79 (m, 1H, H3'b), 4.59-4.55 (m, 4H, 2 x $ArOCH_2$), 4.31-4.24 (m, 1H, H4'a), 4.00-3.50 (m, 14H, H4'b, H5'b, H5''b, H5'a, 2 x OCH_3, 2 x NCH, $POCH_2$), 3.39-3.30 (m, 1H, H5''a), 3.21-3.15 (m, 1H, H2'a), 3.03-2.97 (m, 1H, H2'b), 2.75-2.58 (m, 3H, H2''a, CH_2CN),2.46-2.42 (m, 1H, H2''b), 1.91-1.84 (m, 4H, 2 x CH_2), 1.55-1.45 (m, 6H, $CH_2CH_2CH_2$), 1.20-1.16 (m, 9H, 3 x CH_3), 1.13-1.11 (m, 3H, CH_3), 0.94 (s, 9H, $SiC(CH_3)_3$), 0.90 (s, 9H, $SiC(CH_3)_3$), 0.17 (s, 3H, $SiCH_3$), 0.16 (s, 3H, $SiCH_3$), 0.074 (s, 3H, $SiCH_3$), 0.069 (s, 3H, $SiCH_3$). ^{13}C NMR (125.7 MHz, d_6-acetone, ppm): 166.80, 160.99, 160.97, 158.62, 158.56, 158.20, 152.72, 152.65, 152.60, 151.70, 151.62, 151.57, 145.28, 145.27, 141.10, 141.03, 140.77, 135.95, 135.88, 135.85, 135.82, 130.12, 130.07, 129.97, 129.94, 129.44, 129.41, 128.11, 128.04, 127.53, 126.53, 121.22, 118.71, 118.66, 118.48, 118.11, 117.95, 114.72, 112.86, 112.85, 112.81, 112.80, 87.94, 86.13, 86.03, 85.88, 84.70, 84.64, 84.24, 74.29, 74.15, 73.95, 73.81, 72.41, 67.95, 67.15, 67.10, 64.19, 63.11, 61.85, 58.73, 58.67, 58.58, 58.52, 54.59, 54.57, 47.26, 43.11, 43.02, 39.61, 38.40, 28.82, 25.70, 25.68, 25.45, 25.30, 24.02, 24.00, 23.96, 23.94, 21.90, 21.88, 20.39, 19.87, 19.82, 19.76, 18.94, 18.87, 18.03, 17.67, 13.60, -5.34, -5.44, -6.08, -6.12. ^{31}P NMR (202.3 MHz, d_6-acetone, ppm): 148.42, 148.22. IR (thin film); ν_{max} (cm^{-1}) = 3412, 2963, 2929, 2857, 2367, 2332, 1713, 1607, 1510, 1496, 1464, 1419, 1381, 1364, 1249, 1178, 1080, 1032, 978, 836, 780, 754, 691. HRMS (ESI-MS) *m/z* calculated for $C_{85}H_{113}N_{12}NaO_{15}PSi_2^+$ 1651.7617: found 1651.7664 $[M+Na]^+$

Preparation, purification and characterization of the modified oligonucleotides ***G7*** *and* ***GG7***

Different sequences were used to monitor the repair of alkylation at the O^6-atom of guanine and the O^4-atom of thymine. A DNA sequence with a PvuII cut site was employed to examine the repair of alkylation at the O^6-atom of guanine while a BclI cut site was used to monitor the repair of alkylation at the O^4-atom of thymine. Following the DNA repair reaction, the reaction mix is incubated with the appropriate restriction enzyme and the products analyzed by gel electrophoresis. If an O^6-alkyl guanine or O^4-alkyl

thymine nucleotide is present in the cut site, the restriction enzyme will be inhibited and the full length DNA will be observed on the polyacrylamide gel. If the alkyl adduct is removed by an AGT protein a native guanine/thymine will take its place in the PvuII/BclI cut site allowing DNA cleavage by the restriction enzyme.

The oligonucleotides used as substrates for the repair of alkylated guanine were variations of the following 14-mer, where the underlined sequence represents the PvuII recognition sequence:[3]

5'-GGCTCAG*CTGCCAG-3'; the structures of the alkylated guanines are shown in **Figure 1**.

O^6Me G, G* = O^6-methyl 2'-deoxyguanosine

G7, G* = O^6-heptan-7-ol 2'-deoxyguanosine

G7G, G* = O^6-heptylene-O^6-2'-deoxyguanosine nucleoside

The complementary strand, 5'-CTG GCA GCT GAG CC-3', and the control oligonucleotide (G* = 2'-deoxyguanosine) were synthesized according to standard protocols. **O^6Me G** was prepared by TriLink Biotechnologies (San Diego, CA, USA). Methodology for the preparation of **XLG** has been previously reported.[1,4,5]

The oligonucleotides used as substrates for the repair of alkylated thymine were variations of the following 14-mer, where the underlined sequence represents the BclI recognition sequence:

5'-GGC TT*G ATC ACC AG-3'; the structures of the alkylated thymines are shown in **Figure 1**.

O^4Me T, T* = O^4-methyl thymidine

T4, T* = O^4-butan-4-ol thymidine

T7, T* = O^4-heptan-7-ol thymidine

The complementary strand, 5'-CTG GTG ATC AAG CC-3', and the control oligonucleotide (T* = thymidine) were synthesized according to standard protocols. **O^4Me T** was prepared by TriLink Biotechnologies (San Diego, CA, USA). The syntheses of **T4**, **T7** have been previously described.[6] All oligonucleotides were purified by anion exchange HPLC and their identities confirmed by ESI-MS and enzymatic digestion.

Novel modified **G7** and **GG7** sequences, were assembled with an Applied Biosystems Model 3400 synthesizer on a 1.5 µmol scale using *β*-cyanoethylphosphoramidite chemistry supplied by the manufacturer with slight modifications to coupling times. The nucleoside phosphoramidites protected with standard groups were prepared at a concentration of 0.1 M in anhydrous MeCN for the 3'-*O*-deoxyphosphoramidites, and 0.15 M in anhydrous MeCN for the adducted 3'-*O*-deoxyphosphoramidite. Oligomer sequence assembly was carried out as previously described.[2,7] The capping step of the assembly was carried out using phenoxyacetic anhydride/pyridine/tetrahydrofuran 1:1:8 (v/v/v; solution A) and 1-methyl-imidazole/ tetrahydrofuran 16:84 (w/v; solution B). Coupling wait times for phosphoramidites **3** and **5** were extended to 10 min (compared to 2 min for the commercially available

phosphoramidites). Protecting group removal and cleavage from the solid support was carried out by treatment with NH_4OH(aq) : EtOH (3:1 v/v) for 16 h at 55 ^{o}C in 2 mL screw-cap microfuge tubes fitted with Teflon lined caps. The crude oligomers were transferred and lyophilized in a Speedvac concentrator. Silyl protecting groups of oligomer **G7G** were removed by treatment with NEt_3•3HF (200 µL, pellet initially sonicated (2 x 15 s)) for 24 h at room temperature under gentle rocking. Oligomers were precipitated using cool *n*-butanol (400 µL) and the resulting mixture was allowed to cool to -20°C for 10 min, followed by spinning the samples down. The supernatant was removed and the pellet was washed with another aliquot of *n*-butanol (400 µL). Purification was achieved by strong anion exchange HPLC using a Dionex DNAPAC PA-100 column (0.4 cm x 25 cm) purchased from Dionex Corp, Sunnyvale, CA using a linear gradient of 0-52% buffer B over 24 min (buffer A: 100 mm Tris-HCl, pH 7.5, 10% MeCN and buffer B: 100 mm Tris-HCl, pH 7.5, 10% MeCN, 1M NaCl) at 55 ^{o}C. The columns were monitored at 260 nm for analytical runs or 280 nm for preparative runs. The purified oligomers were desalted using C-18 SEP PAK cartridges (Waters Inc.) as previously described.[8] The molecular mass of the modified oligomers were identified by ESI-MS and the measured values were in agreement with the expected masses (see **Supplementary Figures S9** - **S10** for MS spectra).

UV thermal denaturation

Molar extinction coefficients for the unmodified and cross-linked oligonucleotides were calculated from those of the mononucleotides and dinucleotides using the nearest-neighbor approximations (M^{-1} cm^{-1}). All duplexes were prepared by mixing equimolar amounts of the interacting strands and lyophilizing the mixture to dryness. The resulting pellet was then dissolved in 90 mM sodium chloride, 10 mM sodium phosphate, 1 mM EDTA buffer (pH 7.0) to give a final concentration of 3.5 µM duplex. Prior to the thermal run, samples were degassed by placing them in a speed-vac concentrator for 2 min. Annealing curves were acquired at 260 nm starting at 95 ^{o}C and decreasing temperature at a rate of cooling of 0.5 ^{o}C min^{-1} until 15 ^{o}C, using a Varian CARY Model 3E spectrophotometer fitted with a 6-sample thermostated cell block and a temperature controller. The samples were then denatured by heating from 15 ^{o}C to 95 ^{o}C at an increasing temperature rate of 0.5 ^{o}C min^{-1} to show reversibility. Denaturing data processing was carried out as described by Puglisi and Tinoco[9] and transferred to Microsoft Excel™ for viewing.

Circular dichroism (CD) spectroscopy

Circular dichroism spectra were obtained on a Jasco J-815 spectropolarimeter equipped with a Julaba F25 circulating bath. Samples were allowed to equilibrate for 10 min at 15 ^{o}C in 90 mM sodium chloride, 10 mM sodium phosphate, 1 mM EDTA (pH 7.0), at a final concentration of 3.5 µM. Each spectrum was an average of 5 scans, collecting at a rate of 50 nm min^{-1}, with a bandwidth of 1 nm and sampling wavelength of 0.2 nm using fused quartz cells (Starna 29-Q-10). The CD spectra were recorded from 350 to 220 nm at 15 ^{o}C. The molar ellipticity (ϕ) was calculated from the equation $\phi = \varepsilon/Cl$, where ε is the relative ellipticity (mdeg), C is the molar concentration of oligonucleotides (moles/L), and l is the path length of the cell (cm). The data were processed using software supplied by the manufacturer (JASCO, Inc.) and transferred into Microsoft Excel™ for viewing.

Nucleic acid molecular modeling

Molecular modeling was performed by using the Hyperchem 7.5 software package from Hypercube utilizing the AMBER force field. Hybridized oligomers containing a d(G·C), d(G7·C), and d(G7G·C) base pair were constructed from the nucleic acid template option using a B-form duplex. Duplexes were solvated with water using a periodic box. Standard Amber99 parameters were used with the dielectric set to constant. "One to four scale factors" non-bonded interactions were set to 0.5 (both electrostatic and van der Waals). Cutoffs were applied to "switched" to an outer and inner radius of 14.5 and 10.5 Å. All structures were geometry optimized using Polak-Ribiere conjugate gradient until the RMS gradient was less than 0.1 kcal/(Å mol) using the periodic boundary condition option.

OGT modeling

Homology modeling was performed using MODELLER 9 version 4 (http://salilab.org/modeller/) using PDB ID: 1t38 as template.[10,11] Ligand docking was performed with AutoDock, version 4.2 and AutoDockTools, version 1.5.4.[12] The coordinates of the **G7G** ligand were obtained from the supplementary material from Fang, *et al*.[13] A simplified version of this ligand, consisting of only the dimer (O^6-2'-deoxyguanosine-heptylene-O^6-2'-deoxyguanosine) was used.

Protein expression and purification

Plasmids containing the gene coding for N-terminally His_6-tagged *E. Coli* OGT protein and hAGT were a gift from Dr. Anthony Pegg. Following transformation into XL-1 Blue cells, OGT protein expression was induced with IPTG once the culture reached on OD_{600} of 0.5. Cells were harvested at 8000 x g for 20 min, suspended in lysis buffer (20 mM Tris-HCl, pH 8.0, 250 mM NaCl, 5 mM TCEP and 1 x Complete, EDTA free, protease inhibitor cocktail (Roche)) and lysed by sonication. The N-terminal His-tagged protein was applied to Ni-NTA resin (Qiagen), washed with lysis buffer containing 10 mM imidazole and eluted with lysis buffer containing 200 mM imidazole.[14] Fractions containing protein content were pooled, dialyzed against 50 mM Tris-HCl, pH 7.6, 100 mM NaCl, 0.5 mM TCEP, 0.1 mM EDTA and 10% glycerol overnight and stored at -80˚C. Protein concentration was determined by the absorbance at 280 nm; $\varepsilon = 32555\ M^{-1}cm^{-1}$ (OGT) (calculated with the ProtParam tool (http://ca.expasy.org)). The gene for hAGT was expressed and purified according to the method of Edara[15] and protein content determined by the Bradford assay.[16]

Mutagenesis

Site-directed mutagenesis of the *ogt* gene to produce the S134P variant was performed using the Quick Change method (Stratagene, La Jolla, CA, USA) employing 5'CGGATCGAATCCCATC**CC**CATCGTGGTACCTTGCCATCGGG3' , and 3'GCCTAGCTTAGGGTAG**GG**GTAGCACCATGGAACGGTAGCCC5' as primers. The bold and underlined nucleotides depict the mutations required for the Ser to Pro alteration while the underlined nucleotide was introduced as a silent mutation for diagnostic purposes. Following mutagenesis, the presence of the mutation was confirmed by gene sequencing (BioS&T, Inc. Lachine, Canada) and by mass spectrometry of the purified protein on a Q-TOF instrument (Concordia Centre for Biological Applications of Mass

Spectrometry). The molecular biology and purification procedures for C145S hAGT were conducted as previously described.[1]

Enzymatic Assays

The complementary strands and **XLG** were labelled with ^{32}P, using polynucleotide kinase and [γ-^{32}P]ATP, following the protocol provided by MBI Fermentas (Burlington, Canada) (www.fermentas.com).

The mono-adduct containing dsDNAs were prepared for repair reactions by annealing with their complementary strand, heated to 80˚C and slowly cooled to room temperature to ensure proper duplex formation. Repair reactions were composed of 2 pmol of duplex DNA, 10 pmol of AGT in a total reaction volume of 15 µL in 10 mM Tris-HCl, pH 7.8, 50 mM NaCl, 5 mM EDTA. At each time point the reactions were terminated by incubating the samples at 80˚C for 30 min. $MgCl_2$ was added to the sample to a final concentration of 10 mM, the appropriate restriction enzyme added (4 units of PvuII for ***O*^{6}Me G**, **G7** and **G7G** or 7.5 units of BclI for ***O*^{4}Me T**, **T4** and **T7**) and the sample incubated at 37˚C for 4 h with PvuII or 1 h with BclI. The samples were loaded on a 20% 7 M urea denaturing polyacrylamide gel after the addition of 15 µL of gel loading buffer (89 mM Tris-HCl, 89 mM boric acid, 2 mM EDTA and 80% formamide). The various DNA species were separated by running the gels for 60 min at 400 V using 1 X TBE (89 mM Tris-HCl, 89 mM boric acid, 2 mM EDTA) as the gel running buffer. Radioactive counts were obtained by exposing the gels to a phosphorimaging screen and quantifying the scanned bands using ImageQuant (GE HealthcareLife Sciences, Buckinghamshire, UK).

Repair of **XLG** was carried out at 37˚C for 2 h with 2 pmol of DNA and 100 pmol of AGT in a 15 µL reaction composed of 10 mM Tris-HCl, pH 7.8, 50 mM NaCl, 5 mM EDTA. The reactions were terminated, loaded on a gel and the species quantified as explained for the mono-adduct repair with the omission of $MgCl_2$ and the restriction digestion step.

Binding Assays

Electrophoretic mobility shift assays (EMSA) were carried out using 0.5 nM DNA duplex and increasing concentrations of protein in a 20 µL reaction consisting of 10 mM Tris-HCl, pH 7.6, 5 mM DTT, 10% glycerol, 0.1 mg/ml BSA and 0.1 mM EDTA. Samples were equilibrated at room temperature for 30 min and loaded onto native 10 % polyacrylamide gels (acrylamide:bis-acrylamide = 75:1). Following electrophoresis in 10 mM Tris-acetate, pH 7.6 containing 0.25 mM EDTA, the gels were processed for visualization and quantitation as described above. The C145S variant of hAGT was used for the EMSA to avoid substrate repair during sample equilibration and electrophoresis. Data processing was performed as previously described.[1]

Identification of repair product (DPC) from hAGT-mediated repair of GG7 ssDNA

300 pmol of hAGT was incubated with 300 pmol of **G7G** in 25 µL of Activity Buffer [10 mM Tris-HCl (pH 7.6), 100 mM NaCl and 1 mM DTT] for 30 min at 37 °C. A reaction aliquot (5 µL) was diluted in aqueous 0.1 % (v / v) formic acid subject to HPLC (Agilent 1200 Series system) using a Grace™ Vydac™ C4 (214MS) column (Fisher Scientific) (100mm x 2.1 mm) operated at a flow rate of 0.25 mL/min at room

temperature (22 ^{o}C) with the following gradient method: 0 - 0.1 min, linear gradient from 10 - 20 % B, 0.1 - 6 min, linear gradient from 20 - 95 % B, 6 - 8 min, hold at 95 % B, 8 - 10 min, linear gradient from 95 - 10 % B, 10 - 19 min, hold at 10 % B (buffer A, 0.1% formic acid in water and buffer B, 0.1% formic acid in acetonitrile). The LC system was interfaced to a Micromass Q-Tof Ultima API equipped with an electrospray source set with the following conditions: source voltage 3.5 kV, mass range of 700−1999 m/z in positive ion mode. The theoretical mass of the hAGT-DNA species were calculated by summing the mass of hAGT (21876 Da) and a heptylene linked dG nucleoside (363 Da). The expected masses are reported in **Figure 6** of the main text. If the repair event had occurred at the free nucleoside residue, DPC masses would have been significantly different, which was not observed.

Supplementary Figure S1 - 500 MHz ^{1}H NMR spectrum of compound (**2**) (in $CDCl_3$)

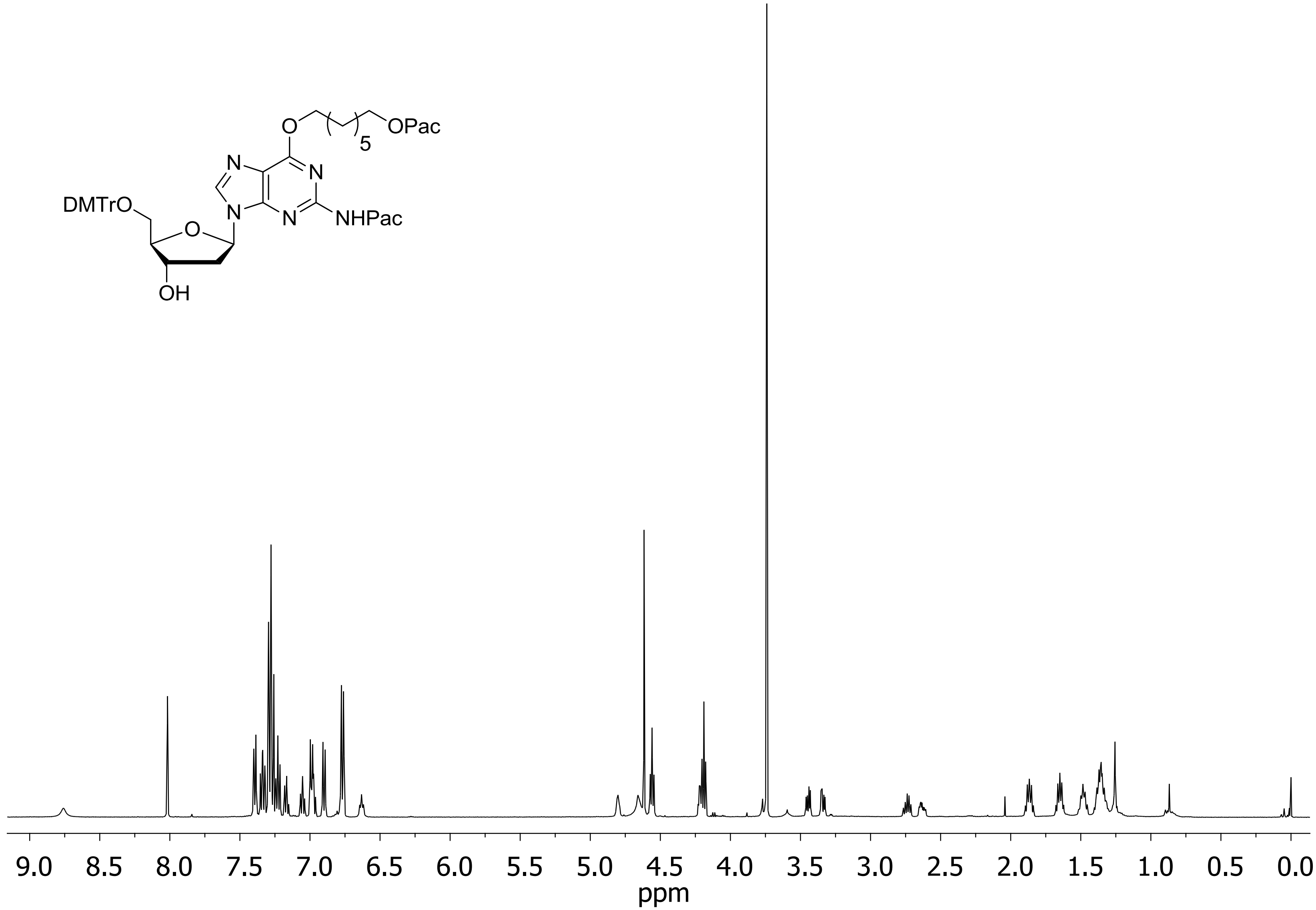

Supplementary Figure S2 - 125.7 MHz ^{13}C NMR spectrum of compound (**2**) (in $CDCl_3$)

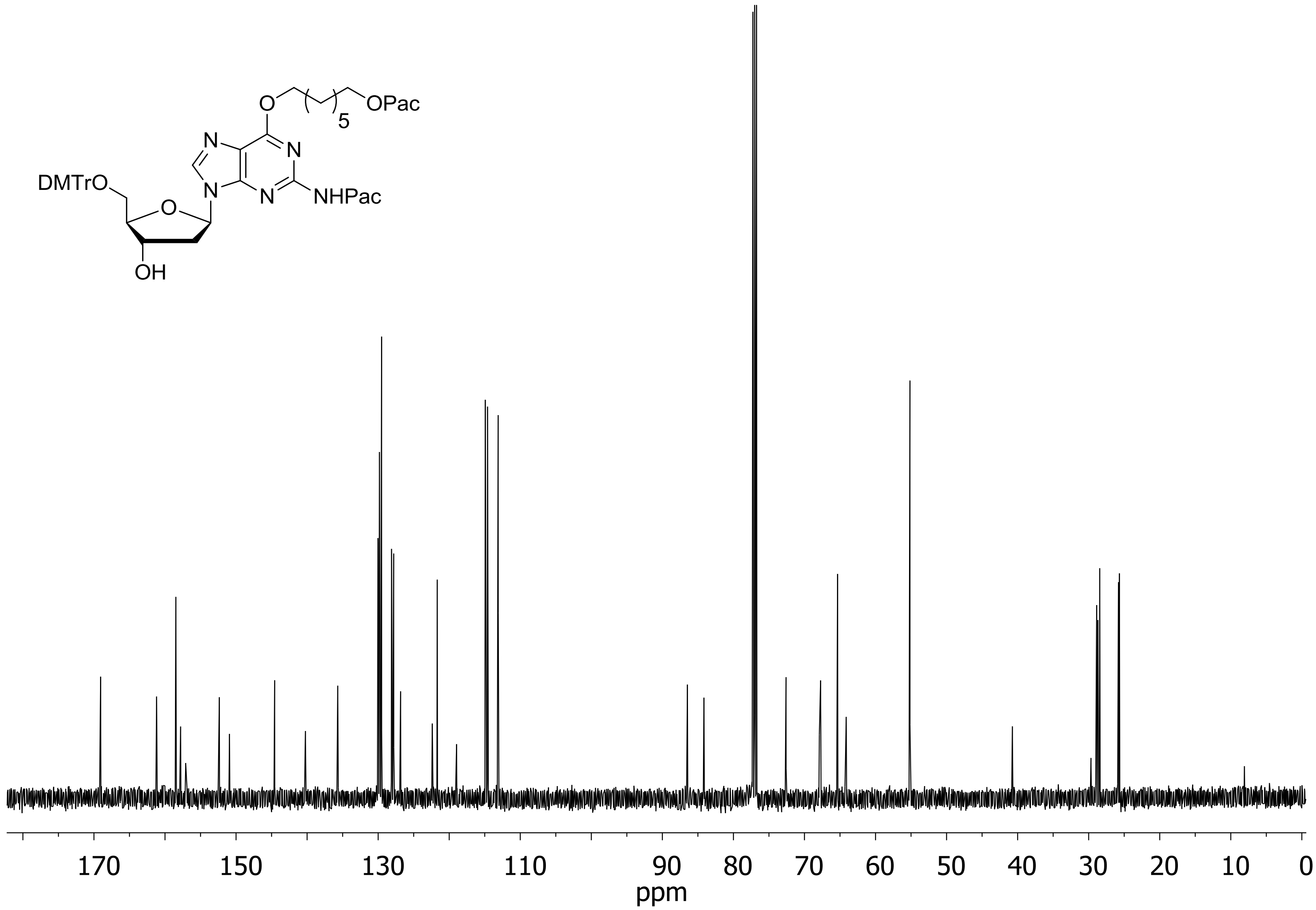

Supplementary Figure S3 - 500.0 MHz ^{1}H NMR spectrum of compound (**3**) (in d_6-acetone)

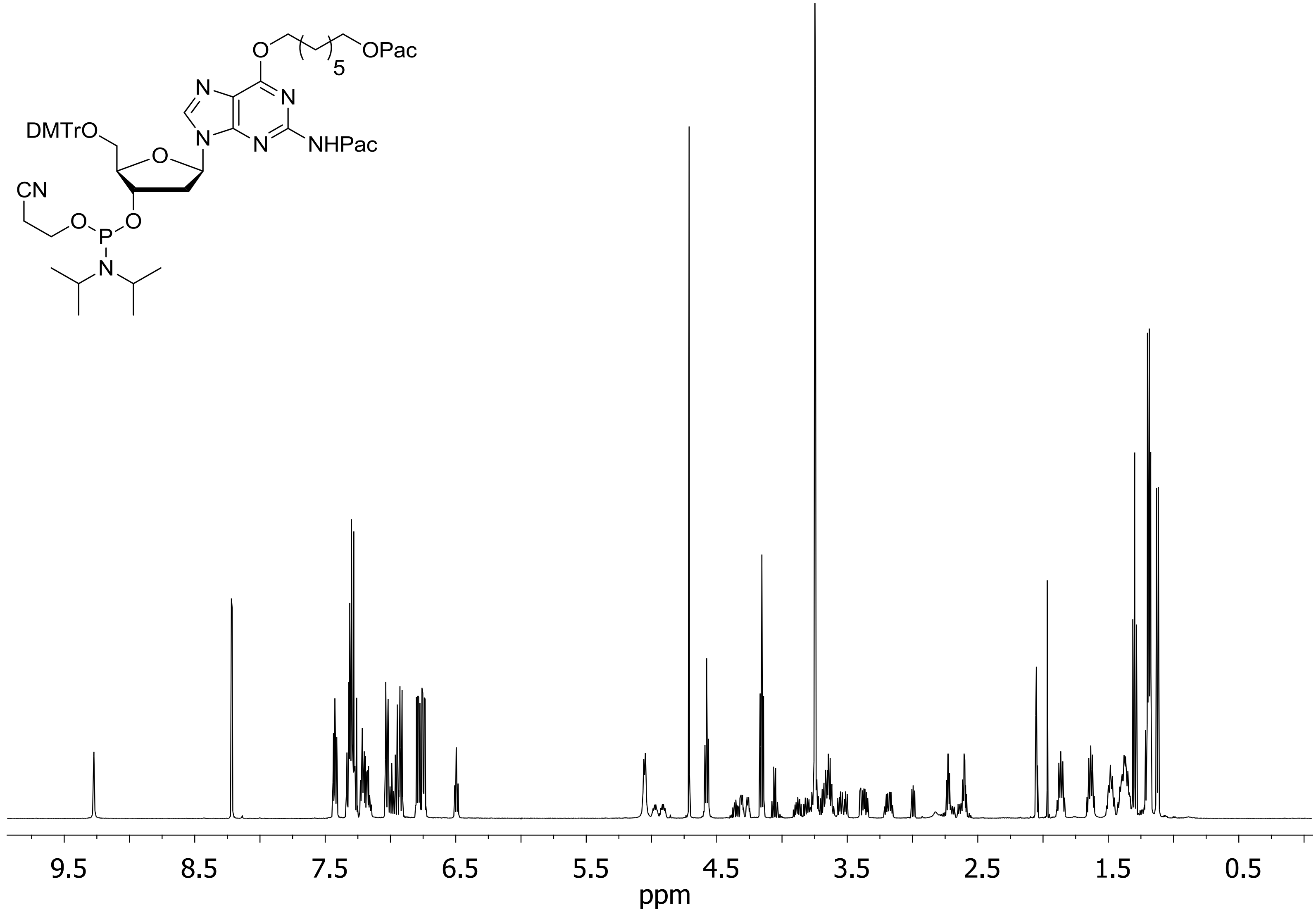

Supplementary Figure S4 - 125.7 MHz ^{13}C NMR spectrum of compound (**3**) (in d_6-acetone)

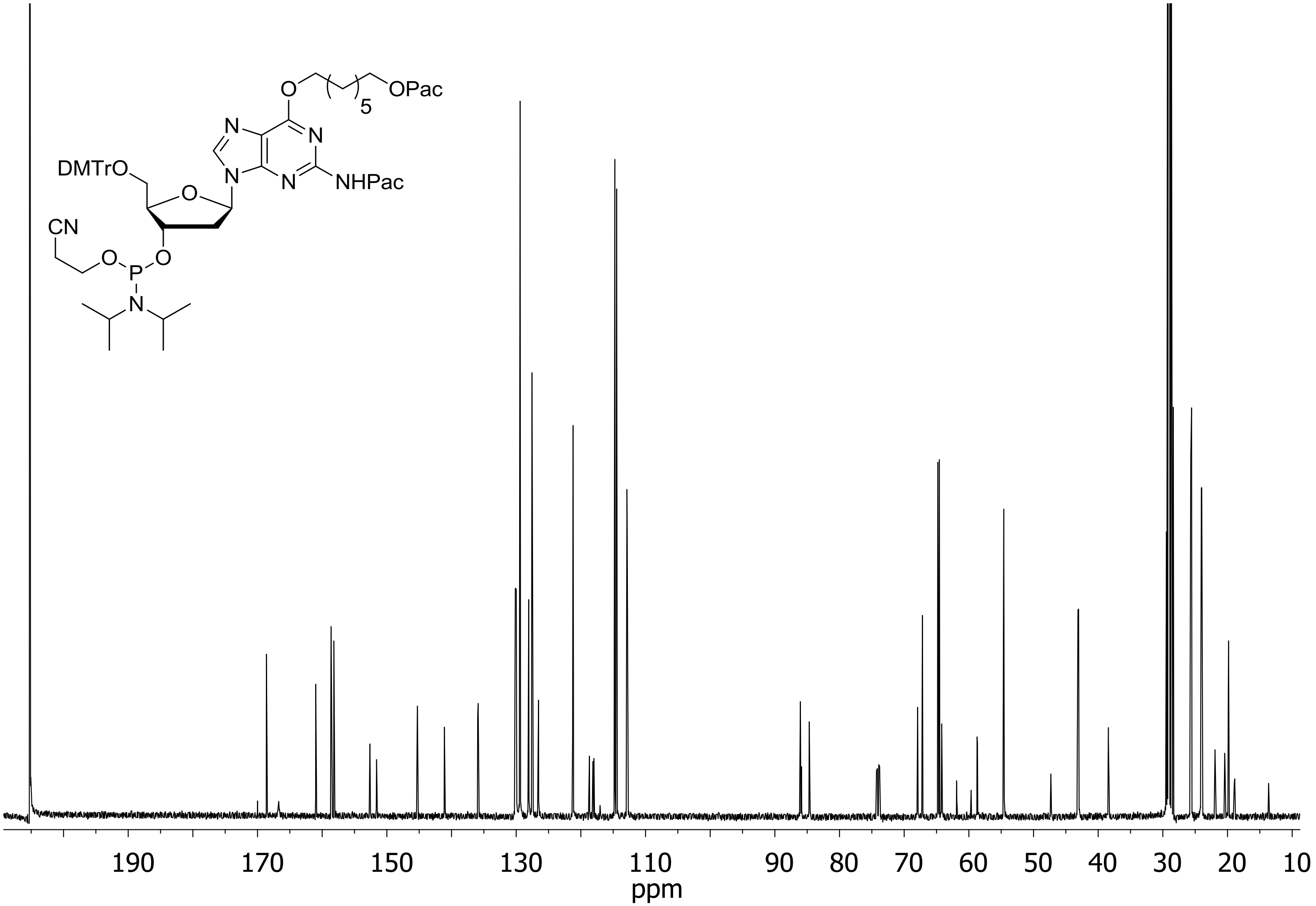

Supplementary Figure S5 - 202.3 MHz ^{31}P NMR spectrum of compound (**3**) (in d_6-acetone)

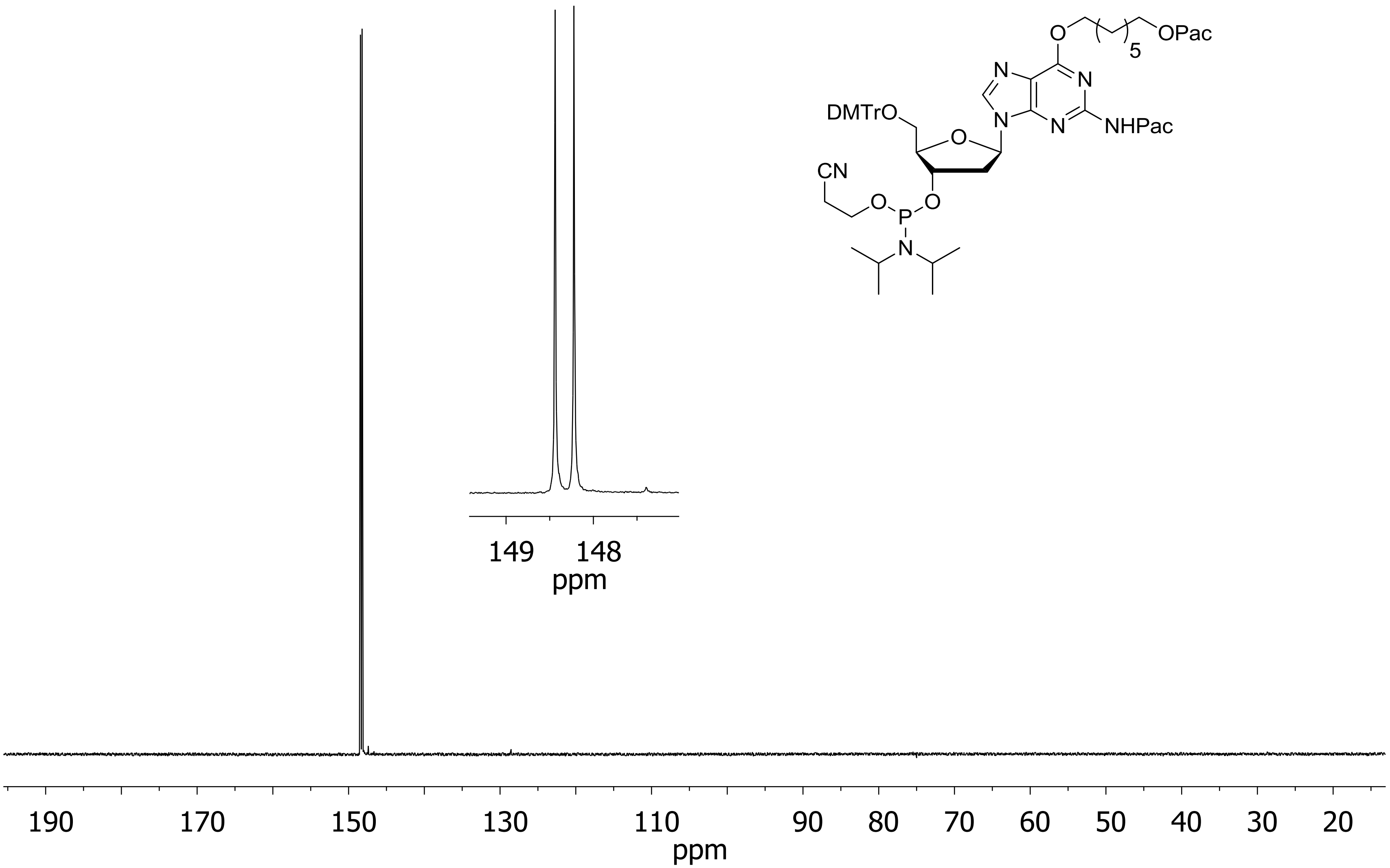

Supplementary Figure S6 - 500.0 MHz ^{1}H NMR spectrum of compound (**5**) (in d_6-acetone)

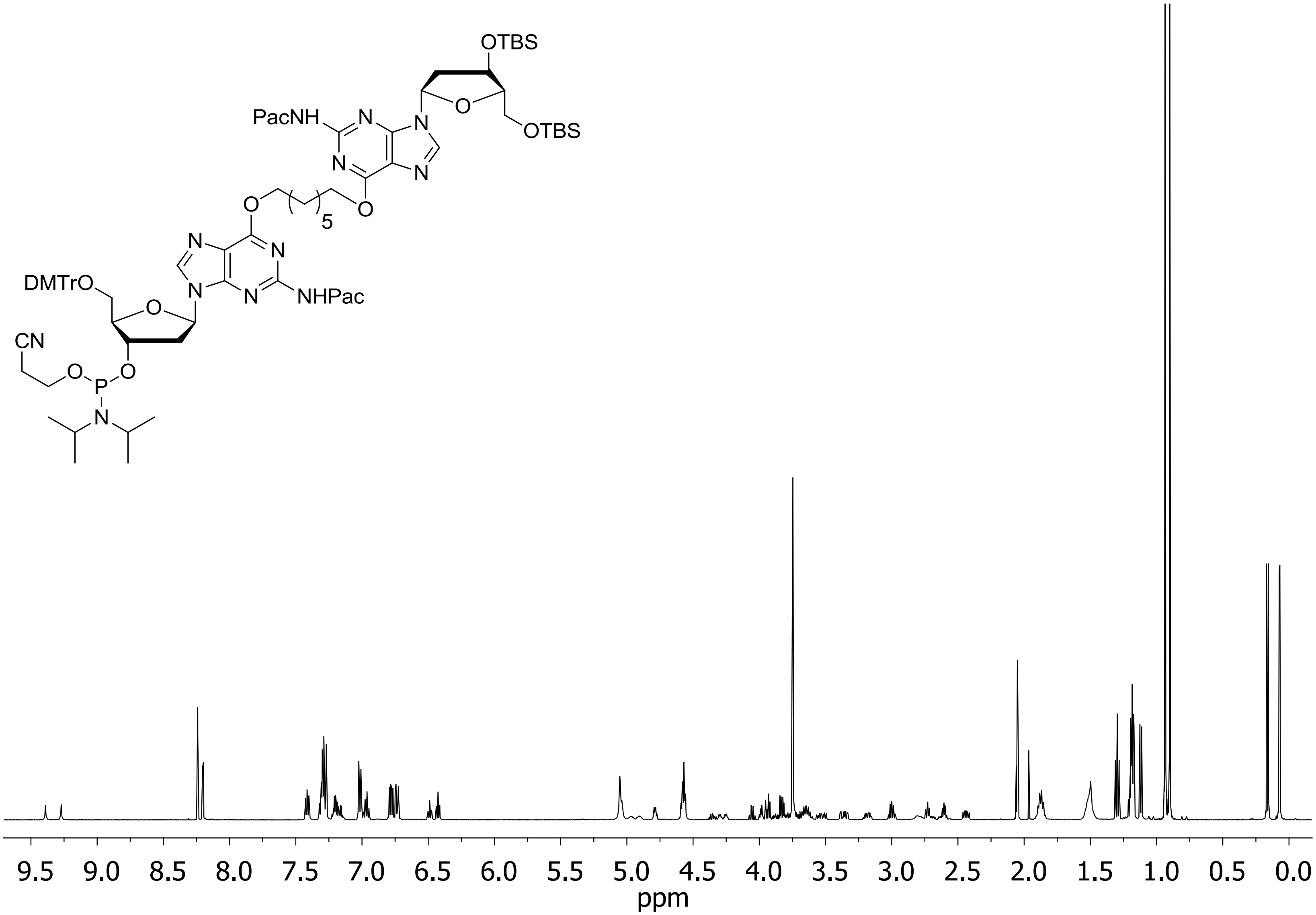

Supplementary Figure S7 - 125.7 MHz ^{13}C NMR spectrum of compound (**5**) (in d_6-acetone)

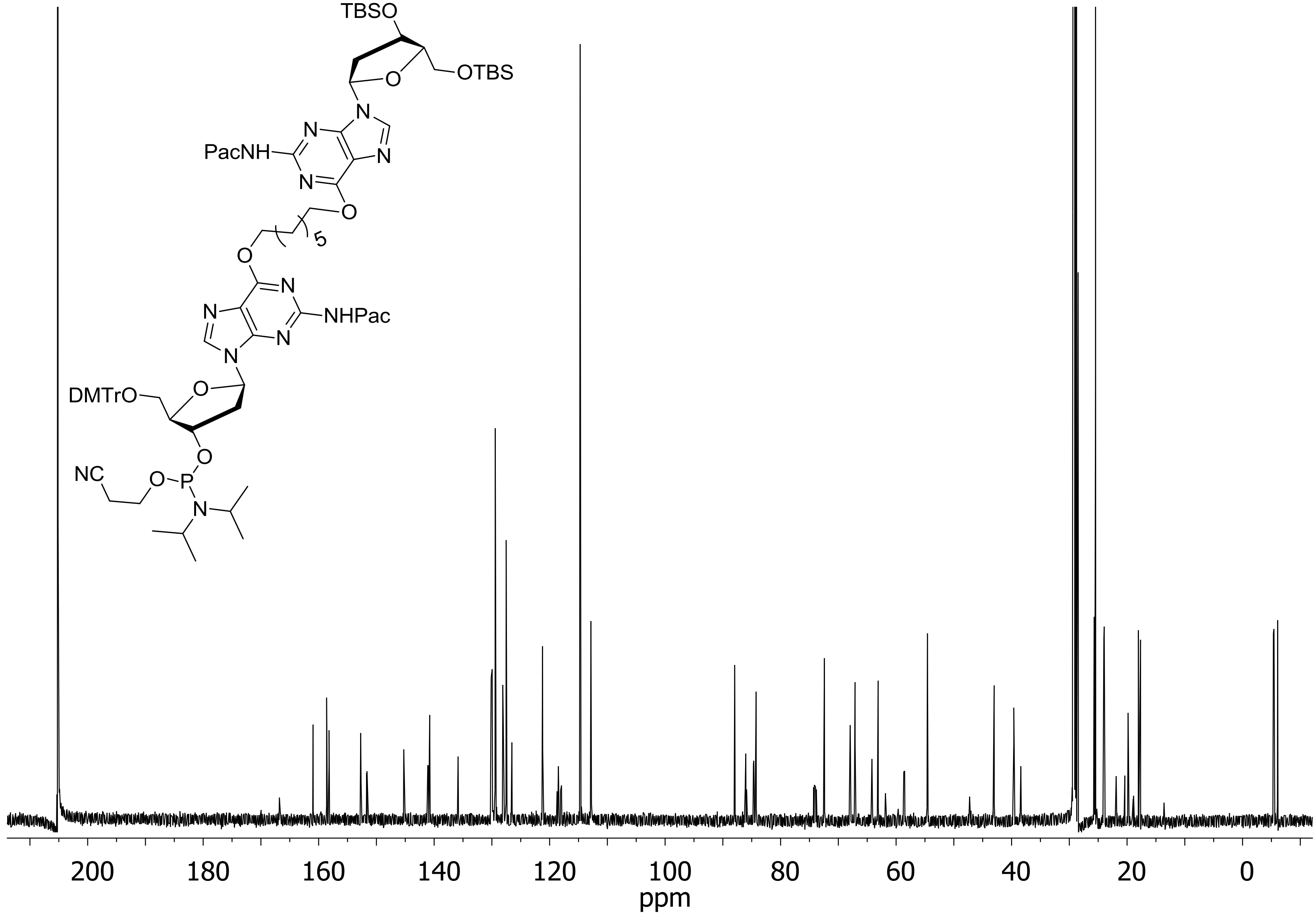

Supplementary Figure S8 - 202.3 MHz ^{31}P NMR spectrum of compound (**5**) (in d_6-acetone)

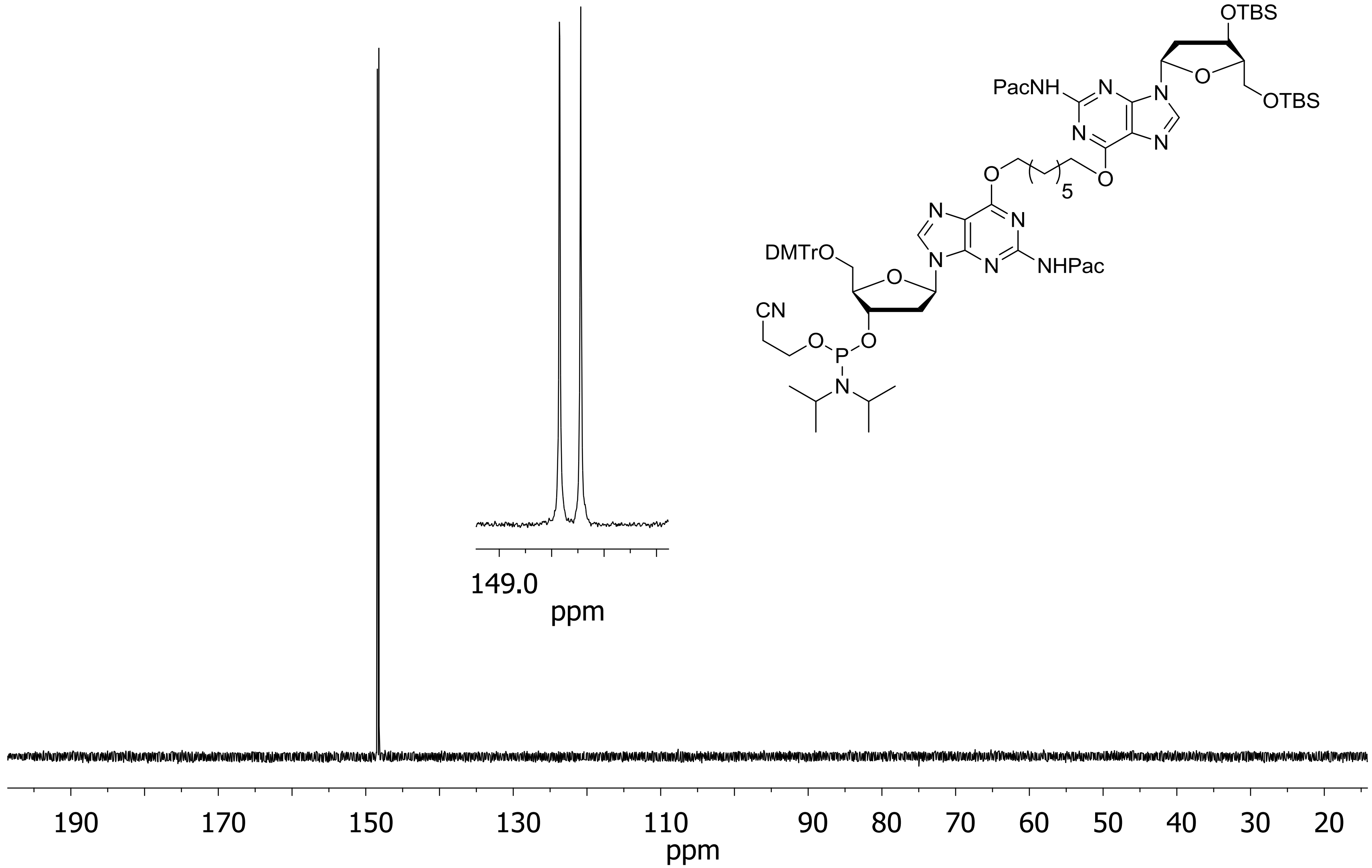

Supplementary Figure S9 - ESI MS spectrum of oligonucleotide **G7** (expected mass of 4378.8)

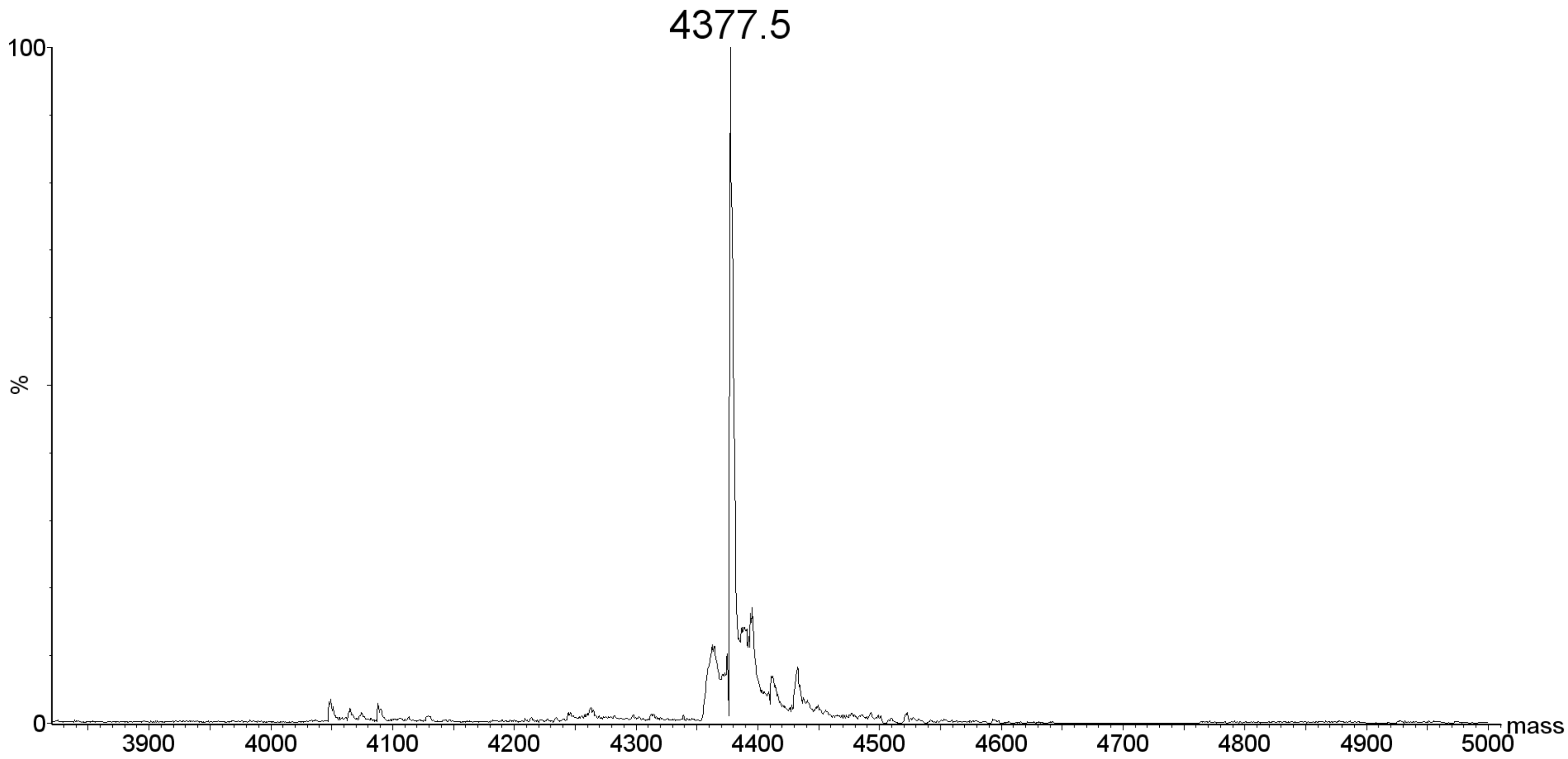

Supplementary Figure S10 - ESI MS spectrum of oligonucleotide **G7G** (expected mass of 4628.2)

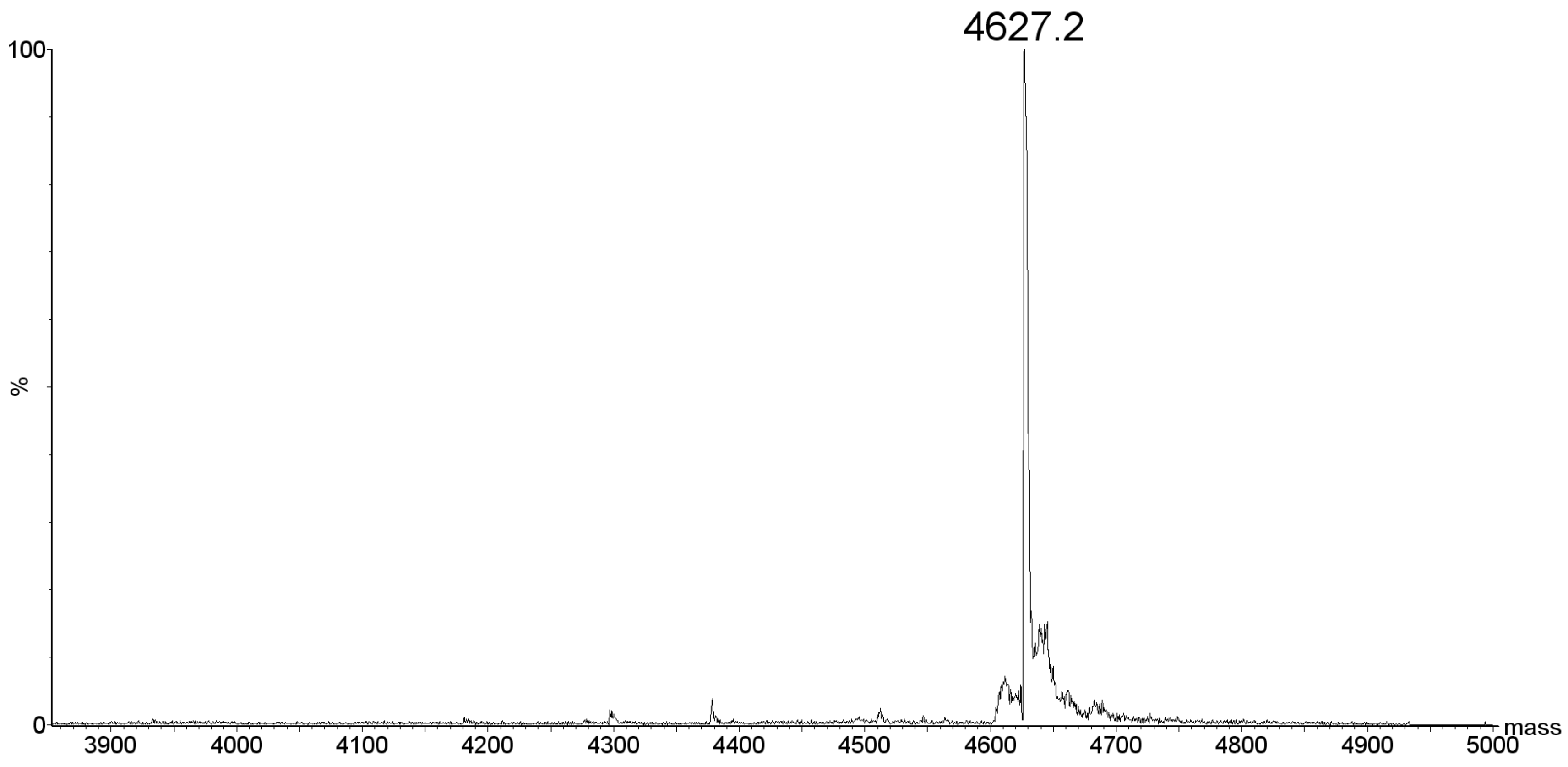

Supplementary Figure S11 - Molecular models of unmodified control (**G**) duplex and duplexes containing **G7** and **G7G** that were geometry optimized using the AMBER forcefield. The modification positions are highlighted in violet.

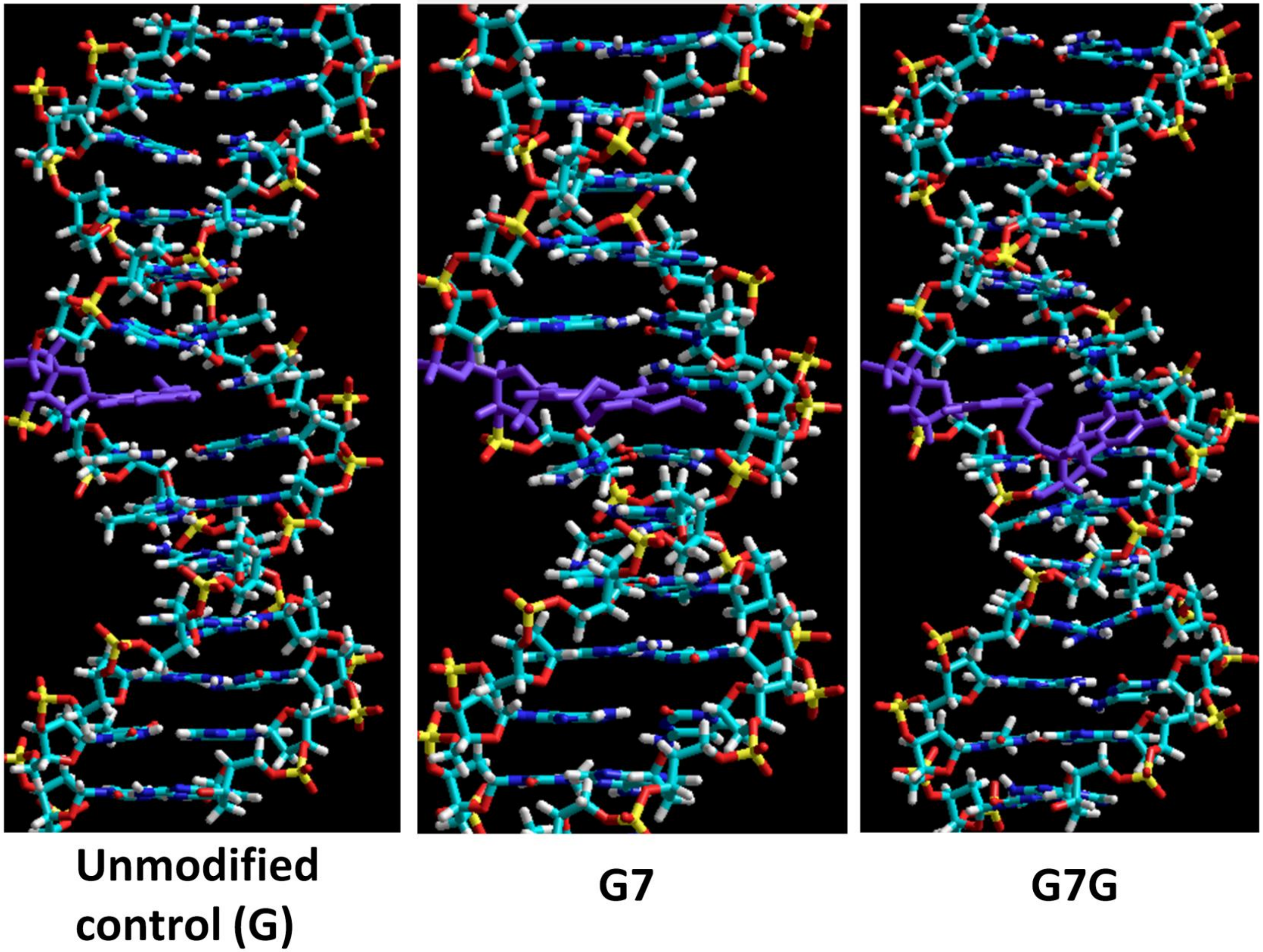

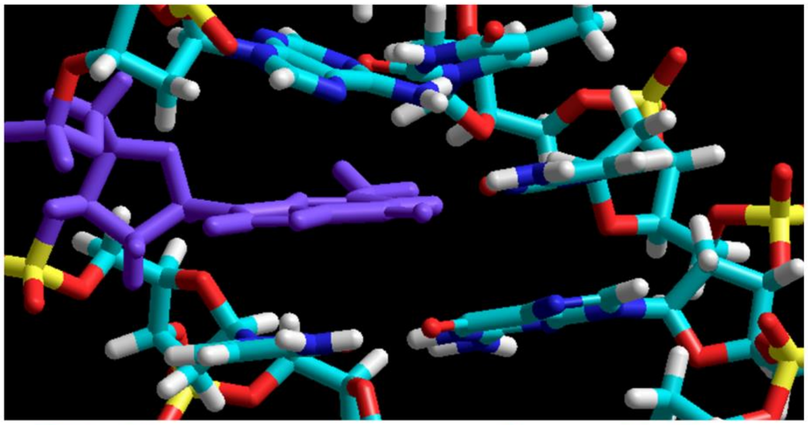

Unmodified control (G)

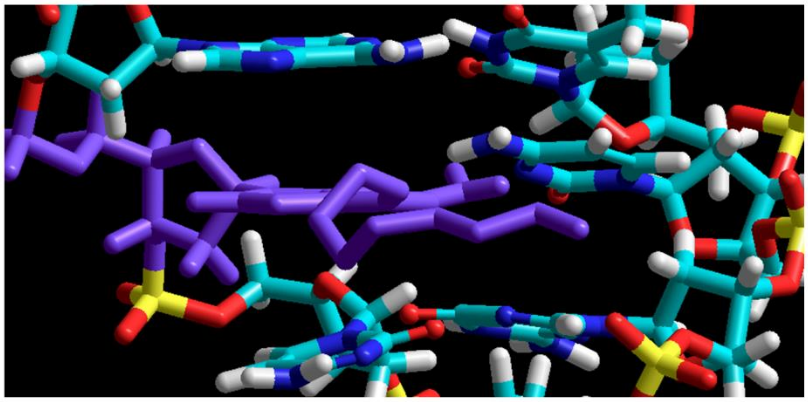

G7

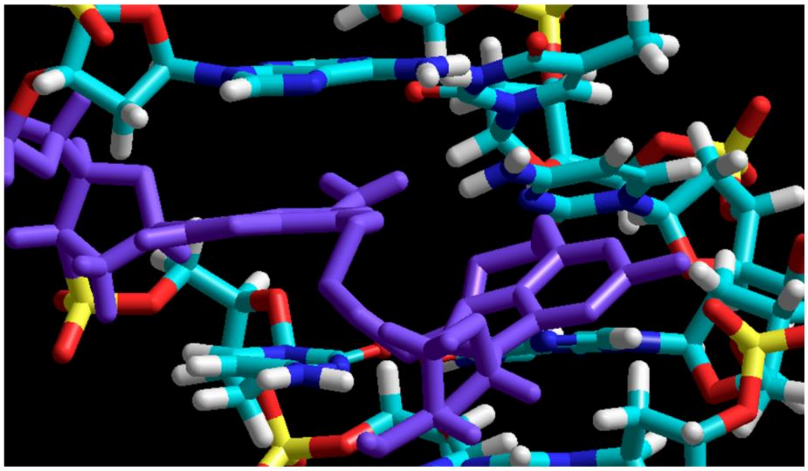

G7G

Supplementary Figure S12 - Time course repair gel of duplexes containing **G7** by (**A**) hAGT**,** (**B**) OGT, or (**C**) OGT S134P. Denaturing gel of the repair of 2 pmol of **G7** by 10 pmol (**A**) hAGT**,** (**B**) OGT, or (**C**) OGT S134P as a function of time: lane 1, 2 pmol Control; lanes 2-8, 2 pmol **G7** + 10 pmol AGT incubated for 2, 7.5, 15, 30, 45, 60, 120 min, respectively.

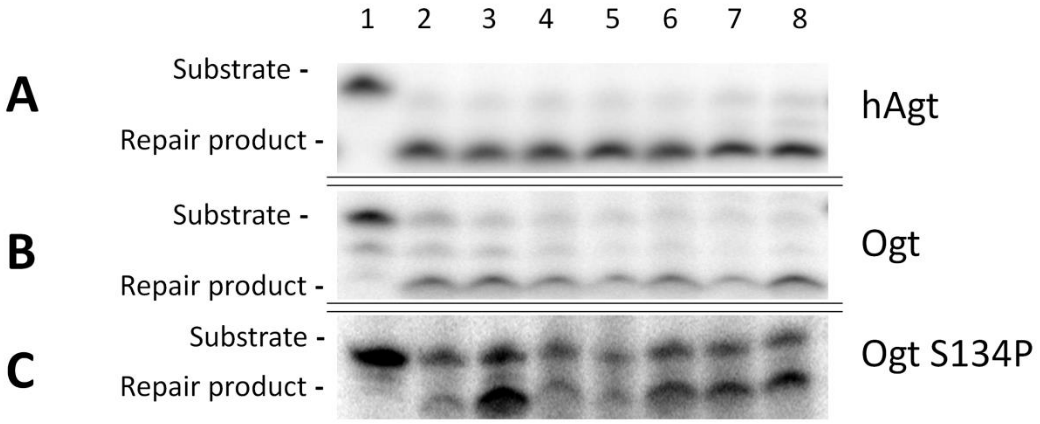

Supplementary Figure S13 - Time course repair gel of duplexes containing **G7G** by (**A**) hAGT**,** (**B**) OGT, or (**C**) OGT S134P. Denaturing gel of the repair of 2 pmol of **G7G** by 10 pmol (**A**) hAGT**,** (**B**) OGT, or (**C**) OGT S134P as a function of time: lane 1, 2 pmol Control; lanes 2-8, 2 pmol **G7** + 10 pmol AGT incubated for 7.5, 15, 30, 45, 60, min, respectively.

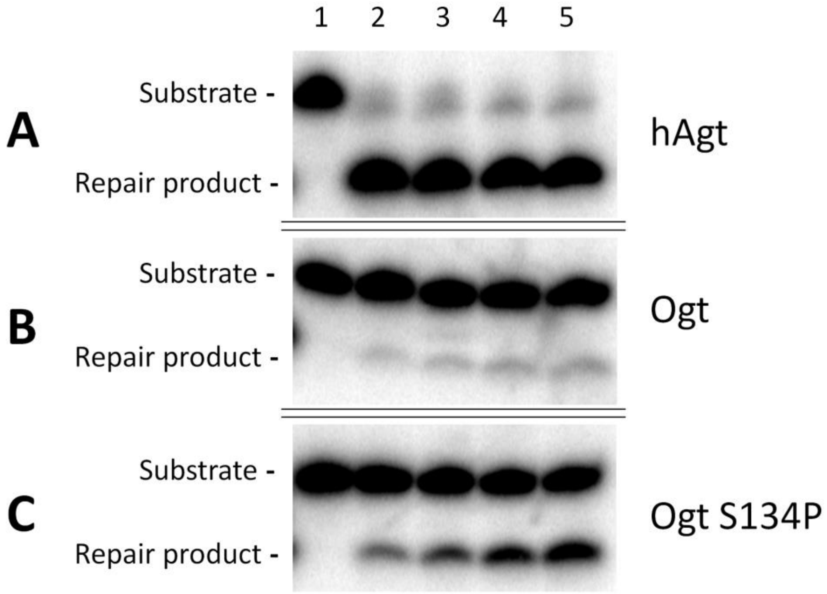

Supplementary References

(1) McManus, F. P., Fang, Q., Booth, J. D. M., Noronha, A. M., Pegg, A. E., and Wilds, C. J. (2010) Synthesis and characterization of an O6-2'-deoxyguanosine-alkyl-O6-2'-deoxyguanosine interstrand cross-link in a 5'-GNC motif and repair by human O6-alkylguanine-DNA alkyltransferase. *Org. Biomol. Chem. 8*, 4414–4426.

(2) O'Flaherty, D. K., and Wilds, C. J. (2016) O6-Alkylguanine DNA alkyltransferase repair activity towards intrastrand cross-linked DNA is influenced by the internucleotide linkage. *Chem. - An Asian J. 11*, 576–583.

(3) Moser, A. M., Patel, M., Yoo, H., Balis, F. M., and Hawkins, M. E. (2000) Real-time fluorescence assay for O6-alkylguanine-DNA alkyltransferase. *Anal. Biochem. 281*, 216–222.

(4) Wilds, C. J., Booth, J. D. M., and Noronha, A. M. (2011) Synthesis of building blocks and oligonucleotides with {G}O6-alkyl-O6{G} cross-links. *Curr. Protoc. Nucleic Acid Chem 44*, 5.9.1–5.9.19.

(5) Wilds, C. J., Booth, J. D., and Noronha, A. M. (2006) Synthesis of oligonucleotides containing an O6-G-alkyl-O6-G interstrand cross-link. *Tetrahedron Lett. 47*, 9125–9128.

(6) McManus, F. P., O'Flaherty, D. K., Noronha, A. M., and Wilds, C. J. (2012) O4-alkyl-2'-deoxythymidine cross-linked DNA to probe recognition and repair by O6-alkylguanine DNA alkyltransferases. *Org. Biomol. Chem. 10*, 7078–7090.

(7) O'Flaherty, D. K., and Wilds, C. J. (2015) Synthesis, characterization, and repair of a flexible O6-2'-deoxyguanosine-alkylene-O6 -2'-deoxyguanosine intrastrand cross-link. *Chem. Eur. J. 21*, 10522–10529.

(8) Noll, D. M., Noronha, A. M., and Miller, P. S. (2001) Synthesis and characterization of DNA duplexes containing an N4C-ethyl-N4C interstrand cross-link. *J. Am. Chem. Soc. 123*, 3405–3411.

(9) Puglisi, J. D., and Tinoco, I. J. (1989) Absorbance melting curves of RNA. *Methods Enzym. 180*, 304–325.

(10) Daniels, D. S., Woo, T. T., Luu, K. X., Noll, D. M., Clarke, N. D., Pegg, A. E., and Tainer, J. A. (2004) DNA binding and nucleotide flipping by the human DNA repair protein AGT. *Nat. Struct. Mol. Biol. 11*, 714–720.

(11) A., S., and T. L., B. (1993) Comparative protein modelling by satisfaction of spatial restraints. *J. Mol. Biol. 234*, 779–815.

(12) Morris, G. M., Goodsell, D. S., Halliday, R. S., Huey, R., Hart, W. E., Belew, R. K., and Olson, A. J. (1998) Automated Docking Using a Lamarckian Genetic Algorithm and an Empirical Binding Free Energy Function. *J. Comput. Chem. 19*, 1639–1662.

(13) Fang, Q., Noronha, A. M., Murphy, S. P., Wilds, C. J., Tubbs, J. L., Tainer, J. A., Chowdhury, G., Guengerich, F. P., and Pegg, A. E. (2008) Repair of O6-G-alkyl-O6-G interstrand cross-links by human O6-alkylguanine-DNA alkyltransferase. *Biochemistry 47*, 10892–10903.

(14) Goodtzova, K., Kanugula, S., Edara, S., Pauly, G. T., Robert, C., Pegg, A. E., and Moschel, R. C. (1997) Repair of O6 -Benzylguanine by the Escherichia coli Ada and Ogt and the Repair of O6 -Benzylguanine by the Escherichia coli Ada and Ogt and the Human O 6 -Alkylguanine-DNA Alkyltransferases. *J Biol. Chem. 272*, 8332–8339.

(15) Edara, S., Kanugula, S., Goodtzova, K., and Pegg, A. E. (1996) Resistance of the Human O 6 -Alkylguanine-DNA Alkyltransferase Containing Arginine at Codon 160 to Inactivation by O 6 -

Benzylguanine. *Cancer Res. 56*, 5571–5575.

(16) Bradford, M. M. (1976) A rapid and sensitive method for the quantitation of microgram quantities of protein utilizing the principle of protein-dye binding. *Anal. Biochem. 72*, 248–254.
